# Nutrient-sensitive protein O-GlcNAcylation shapes daily biological rhythms

**DOI:** 10.1098/rsob.220215

**Published:** 2022-09-14

**Authors:** Xianhui Liu, Joanna C. Chiu

**Affiliations:** ^1^ Department of Entomology and Nematology, College of Agricultural and Environmental Sciences, University of California Davis, Davis, CA, USA; ^2^ Department of Pharmacology, School of Medicine, University of California Davis, Davis, CA, USA

**Keywords:** O-GlcNAcylation rhythm, metabolic input, circadian input, biological rhythms, phosphorylation, hexosamine biosynthetic pathway

## Abstract

O-linked-*N*-acetylglucosaminylation (O-GlcNAcylation) is a nutrient-sensitive protein modification that alters the structure and function of a wide range of proteins involved in diverse cellular processes. Similar to phosphorylation, another protein modification that targets serine and threonine residues, O-GlcNAcylation occupancy on cellular proteins exhibits daily rhythmicity and has been shown to play critical roles in regulating daily rhythms in biology by modifying circadian clock proteins and downstream effectors. We recently reported that daily rhythm in global O-GlcNAcylation observed in *Drosophila* tissues is regulated via the integration of circadian and metabolic signals. Significantly, mistimed feeding, which disrupts coordination of these signals, is sufficient to dampen daily O-GlcNAcylation rhythm and is predicted to negatively impact animal biological rhythms and health span. In this review, we provide an overview of published and potential mechanisms by which metabolic and circadian signals regulate hexosamine biosynthetic pathway metabolites and enzymes, as well as O-GlcNAc processing enzymes to shape daily O-GlcNAcylation rhythms. We also discuss the significance of functional interactions between O-GlcNAcylation and other post-translational modifications in regulating biological rhythms. Finally, we highlight organ/tissue-specific cellular processes and molecular pathways that could be modulated by rhythmic O-GlcNAcylation to regulate time-of-day-specific biology.

## Graphical abstract

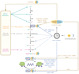

## 1. Introduction

Organisms from all domains of life exhibit daily biological rhythms to adapt to changes in their environment over the 24 h day–night cycle. In animals, daily rhythms of physiology, metabolism and behaviour are strongly regulated by the circadian clock, an endogenous biological timer that enables animals to anticipate predictable changes in biotic and abiotic factors [1,2]. The circadian clock is a molecular oscillator that relies on transcriptional–translational feedback mechanisms operated by key clock transcription factors to generate daily oscillations in gene expression. In coordination with processes that are regulated by post-transcriptional mechanisms, clock-regulated rhythmic gene expression programs that are often tissue- and cell-specific produce daily rhythms in clock outputs. The outputs of animal circadian clocks are all-encompassing and include rhythmic processes such as sleep–wake cycles, feeding–fasting cycles, metabolism, hormone production and secretion, immune response, neuronal excitability and even permeability of the blood–brain barrier [3–10]. There is growing evidence that some clock outputs are themselves zeitgebers (i.e. time-givers) and can feedback to the molecular oscillator to reinforce and/or modulate daily biological rhythms. The feeding–fasting cycle is one such clock output and studies have shown that key clock transcription factors that form the core of the molecular oscillator can be regulated by metabolites or nutrient-sensitive hormones, such as heme, NAD/NADH (nicotinamide adenine dinucleotide/reduced form of nicotinamide adenine dinucleotide), AMP/ATP (adenosine monophosphate/adenosine triphosphate), acetyl coenzyme A, glucocorticoids and glucagon (reviewed in [11]).

Besides impacting daily biological rhythms by modulating the activities of key clock transcription factors, metabolic feedback from feeding–fasting cycles can also regulate daily rhythms through other mechanisms beyond the circadian clock. For example, feeding–fasting cycles can drive rhythmic production of NAD^+^, which serves as coenzyme for histone deacetylases class III, also known as sirtuins, to regulate daily rhythmicity in epigenomic landscape and global gene expression [12,13]. Feeding activity also contributes to daily oscillation of protein translation [14]. This was shown to be mediated by the nutrient-sensitive mTOR pathway, amino acid sensing pathways and metabolic modification of mRNA.

We recently established that integration of circadian signals and rhythmic metabolic input can regulate daily cellular physiology through rhythmic protein O-linked-*N*-acetylglucosaminylation (O-GlcNAcylation) [15]. O-GlcNAcylation has the potential to modify the function of thousands of proteins [16–19], and has been shown to play a critical role in maintaining animal circadian rhythms [20–23]. Furthermore, since both O-GlcNAcylation and phosphorylation modify serine and threonine residues [16,17,24], rhythmic O-GlcNAcylation may contribute to robust oscillation of the 24 h phosphoproteome and regulate its time-of-day specific function [25–28]. These post-translational mechanisms could bypass regulation at the transcriptional level to directly modulate protein function in a time-specific and nutrient-sensitive manner. Interestingly, our studies showed that the amplitude of daily protein O-GlcNAcylation rhythm is severely dampened if animals are fed at an unnatural time window (i.e. time of day at which they are normally fasting [15]). This suggests that rhythmic functions of cellular proteins could be impaired by mistimed meals, a common occurrence in modern society. Our findings point to the likelihood that the beneficial effects of time-restricted feeding [29–35], a practice that limits food consumption to 8–12 h during an individual's natural active period and has been shown to maintain robust circadian rhythms, enhance health span and alleviate metabolic diseases, may be partially mediated via daily O-GlcNAcylation rhythm.

In the remainder of the review, we will summarize the regulation of daily rhythmicity in O-GlcNAcylation by metabolic and circadian signals, outline interactions between O-GlcNAcylation and other post-translational modifications (PTMs), and highlight cellular processes that are potentially regulated by rhythmic O-GlcNAcylation.

## 2. Regulation of O-GlcNAcylation in the context of daily biological rhythm

**Figure 1.**
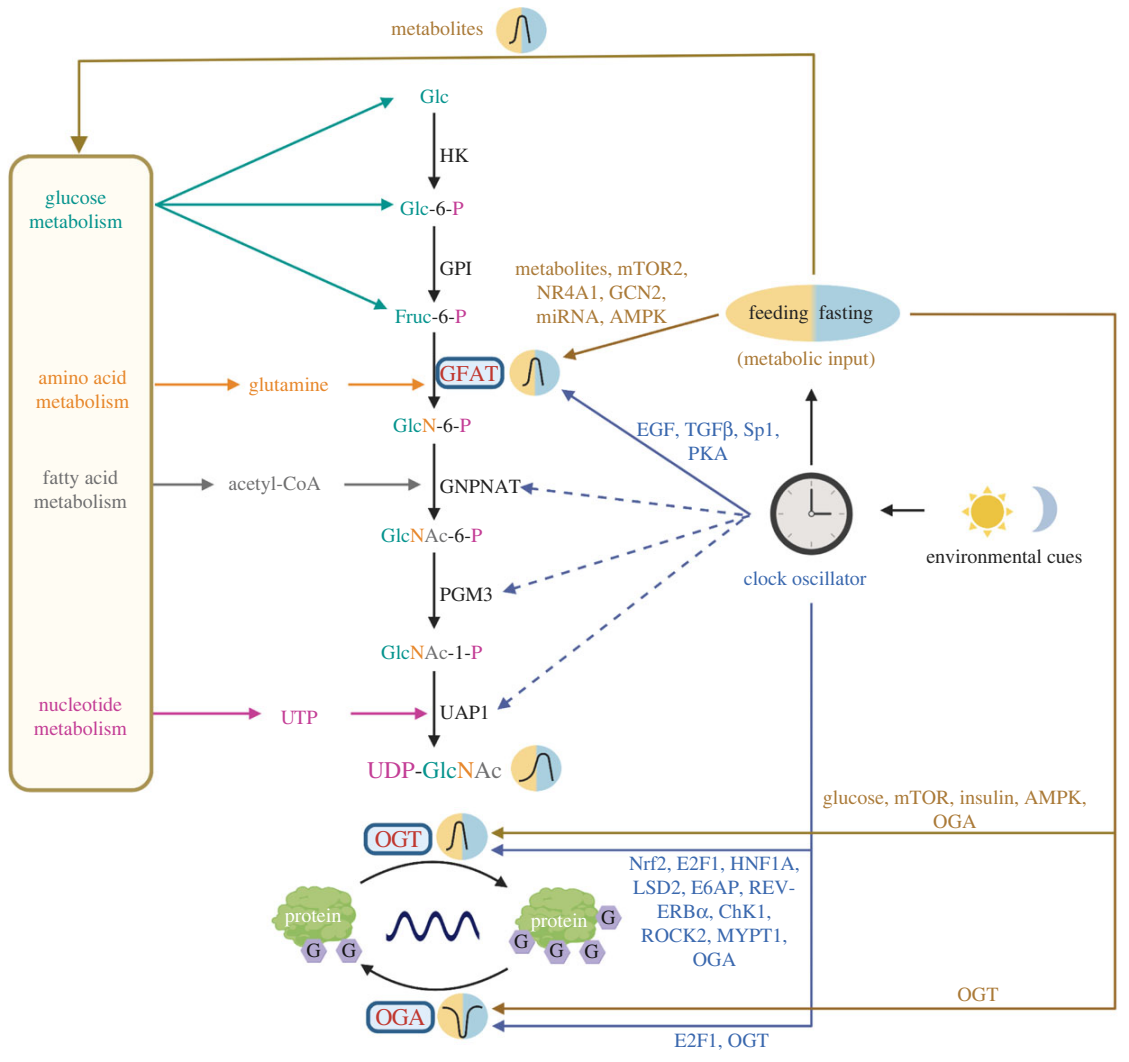
Schematic illustrating metabolic and circadian regulation of rhythmic protein O-linked-*N*-acetylglucosaminylation (O-GlcNAcylation). The circadian clock oscillator receives environmental signals and regulates daily feeding–fasting cycles. Feeding–fasting cycles rhythmically provide input to hexosamine biosynthetic pathway (HBP), which contributes to rhythmic production of UDP-GlcNAc [15]. O-GlcNAc transferase (OGT) takes UDP-GlcNAc as a substrate and transfers GlcNAc onto serine and threonine residues of proteins. This process is recognized as O-GlcNAcylation (O-GlcNAc is depicted as G on protein molecules). Metabolic input can also regulate the O-GlcNAcylation rhythm through modifying the activities of glutamine:fructose-6-phosphate amidotransferase (GFAT) [37–49], OGT [21,50–63] and O-GlcNAcase (OGA) [64]. Additionally, the clock oscillator not only regulates feeding–fasting cycles, but also regulates the expression or enzymatic activities of all the HBP enzymes [39,47,65–76] and O-GlcNAc processing enzymes [62,63,71,72,77–93]. The potential mediating factors of metabolic and circadian inputs are illustrated in the schematic diagram; metabolic inputs are depicted in brown and circadian inputs are depicted in blue. The dashed arrows indicate potential regulation without known mechanisms. HK, Hexokinase; GPI, phosphoglucose isomerase; GFAT, glutamine–fructose-6-phosphate aminotransferase; GNPNAT, glucosamine-phosphate *N*-acetyltransferase; PGM3, phosphoacetylglucosamine mutase; UAP1, UDP-*N*-acetyl glucosamine pyrophosphorylase 1; OGT, O-GlcNAc transferase; OGA, O-GlcNAcase; Glc, glucose; Glc-6-P, glucose-6-phosphate; Fruc-6-P, fructose-6-phosphate; GlcN-6-P, glucosamine-6-phosphate; GlcNAc-6-P, *N*-acetylglucosamine-6-phosphate; GlcNAc-1-P, *N*-acetylglucosamine-1-phosphate; UTP , uridine triphosphate; UDP-GlcNAc, uridine diphosphate *N*-acetylglucosamine; mTOR, mammalian target of rapamycin; NR4A1, nuclear subfamily 4 group A member 1; GCN2, general control nonderepressible2; miRNA, microRNA; AMPK, AMP-activated protein kinase; EGF, epidermal growth factor; TGF*β*_,_ transforming growth factor*β*; Sp1, specificity protein 1; PKA, protein kinase A; Nrf2, nuclear factor E2-related factor-2; E2F1, transcription factor E2F1; HNF1A, hepatocyte nuclear factor 1 homologue A; LSD2, lysine-specific histone demethylase 1B; E6AP, ubiquitin ligase E6AP; ChK1, checkpoint kinase 1; ROCK2, Rho-associated coiled-coil forming protein kinase 2; MYPT1, myosin phosphatase target subunit 1.

Protein O-GlcNAcylation is nutrient-sensitive and is tightly linked to cellular metabolic status. For this reason, the regulation and function of O-GlcNAcylation have been extensively studied in the context of metabolic diseases, specifically diabetes and cancers [17,36]. On the contrary, although metabolism and energy status are highly rhythmic over the day–night cycle, the number of studies on rhythmic O-GlcNAcylation and the consequences of its disruption dwarfs in comparison. The cycling of O-GlcNAc groups on proteins is regulated by the level of UDP-GlcNAc (the substrate) and the activities of two O-GlcNAc processing enzymes, O-GlcNAc transferase (OGT) and O-GlcNAcase (OGA) (figure 1). UDP-GlcNAc is produced from the hexosamine biosynthetic pathway (HBP), which integrates metabolites from glucose metabolism (glucose), amino acid metabolism (glutamine), lipid metabolism (acetyl-CoA) and nucleotide metabolism. In this section, we discuss current findings on the regulation of O-GlcNAcylation under the framework of rhythmic biology over a 24 h day–night cycle.

### 2.1. Regulation of HBP pathway by daily rhythm of nutrient availability

Nutrient availability directly determines the level of building blocks for producing UDP-GlcNAc. Given that there is strong support from metabolomics studies showing that nutrient input correlates with feeding activity [15,94–96], metabolic influx into the HBP is expected to be highly rhythmic over the day–night cycle and probably contributes to daily rhythmicity in O-GlcNAcylation. In studies conducted using cultured cells or tissues, elevated production of UDP-GlcNAc has been shown to correlate with higher nutrient concentration in cell media, including glucose, glutamine, glucosamine (GlcN), acetylglucosamine (GlcNAc), free fatty acids and uridine [37,50,97–105]. However, some glucose starvation studies showed contradictory results; glucose starvation was observed to result in elevation of O-GlcNAcylation level [38,50,51]. These conflicting observations could be explained by divergent properties of cell lines or tissue types. For example, Pham *et al.* [106] showed that O-GlcNAcylation levels in different subtypes of diffuse large B-cell lymphoma cell lines respond differently to glucose deprivation. Additionally, increased O-GlcNAcylation upon glucose starvation could be due to altered levels of OGT, OGA or glutamine:fructose-6-phosphate amidotransferase (GFAT) [38,50,51].

Although cell culture and *ex vivo* studies have firmly established the importance of nutritional regulation of HBP and O-GlcNAcylation, *in vivo* studies especially ones that take into account daily rhythmic biology and feeding–fasting cycles are still limited. In the 1990 s, Hawkins *et al.* [107] showed that continuously infusing lipid emulsion, uridine or GlcN for 7 h increases UDP-GlcNAc levels in rat skeletal muscles. To establish the relationship between feeding activity and levels of HBP metabolites including UDP-GlcNAc, we recently monitored feeding rhythm and HBP metabolites in *Drosophila* flies over a 24 h day–night cycle [15]. We observed strong correlation between fly feeding rhythm and daily rhythms in HBP metabolites in fly body tissues. Significantly, we found that shifting the time of food consumption significantly altered the peak time of HBP metabolite rhythm. In summary, we conclude that HBP metabolites and UDP-GlcNAc level are strongly regulated by clock-controlled feeding–fasting cycle and metabolic input. Whether this phenomenon is consistent in other animals, including nocturnal animals, will need to be explored in future studies.

### 2.2. Daily regulation of HBP enzymes

Besides rhythmic metabolic input into the HBP facilitated by clock-controlled feeding–fasting cycles, rhythmic expression and activity of HBP enzymes could also contribute to daily rhythms in protein O-GlcNAcylation. Searching through CirGRDB, a mammalian circadian transcriptomic database [108] and published *Drosophila* transcriptomic datasets [27,109], we found that transcripts encoding all HBP enzymes oscillate in at least one study. Additionally, data mining in circadian proteomic and phosphoproteomic datasets [25,27,110] revealed that the majority of the HBP enzymes have oscillating protein levels and/or phosphorylation. Our recent study reported that GFAT enzyme activity oscillates over a 24 h cycle in flies and rhythmic GFAT activity is regulated by the integration of metabolic and circadian signals [15]. In this section, we will elaborate on our findings and discuss potential mechanisms mediating daily regulation of HBP enzymes. In particular, we will focus on the regulation of GFAT, the rate-limiting enzyme of HBP and the most well studied of all HBP enzymes (figure 1).

The expression of *gfat* mRNA is highly regulated by nutrient availability and nutrient-sensing pathways. *gfat* has two isoforms in animals, *gfat1* and *gfat2*. Both isoforms encode GFAT enzymes that perform the same catalytic function but have distinct tissue-specific distribution (reviewed in [111]). We showed that the expression of *gfat*2 mRNA in fly body tissues is strongly induced by food consumption in *Drosophila* [15], although we did not explore the mechanisms that mediate the observed induction. In tissue culture, expression of *gfat* mRNA has been shown to be stimulated by various nutrients [37,39–42] and mediated by multiple molecular pathways, including mammalian target of rapamycin2 (mTOR2) [41,43], nuclear subfamily 4 group A member 1 (NR4A1) [40], microRNA (miR)-27b-3p [42] and general control nonderepressible2-activating transcription factor 4 pathway [38]. Expression of *gfat* mRNA can potentially be regulated by clock-controlled factors/processes in addition to feeding–fasting cycles and rhythmic metabolic input. These include angiotensin II [112–114], epidermal growth factor (EGF) [39,65,66], transforming growth factor*β* (TGF*β*) [67–69] and Specificity protein 1 [70–72], all of which are known to influence *gfat* expression.

Beyond transcriptional regulation, GFAT enzyme activity is known to be influenced by PTMs and feedback regulation from HBP metabolites. We reported that the circadian clock strongly regulates daily GFAT activity through unknown post-transcriptional and/or post-translational mechanism(s) [15]. Interestingly, the kinases that have previously been identified to regulate GFAT activities are also known effectors of circadian signals or clock-controlled metabolic signals. These include AMP-activated protein kinase (AMPK) [44,45], mTOR2 [46] and protein kinase A (PKA) [47,73–76]. In particular, PKA-directed phosphorylate site at GFAT1 S235 [74] is shown to oscillate over a circadian cycle in mouse liver [25]. However, the function of GFAT1 pS235 is currently unclear. Finally, glucosamine-6-phosphate (GlcN-6-P) and UDP-GlcNAc, the direct product from the GFAT-catalysed reaction and end product of the HBP respectively, can feedback to inhibit GFAT activity [47–49]. As our study found that GlcN-6-P and UDP-GlcNAc levels oscillate over the day–night cycle with peak time corresponding to feeding period [15], HBP metabolites likely represent important signals to shape daily GFAT activity.

In summary, as we concluded in our studies in *Drosophila* [15], the HBP enzyme GFAT represents an important integration hub of circadian and metabolic signals to regulate the production of UDP-GlcNAc and cellular protein O-GlcNAcylation.

### 2.3. Daily regulation of O-GlcNAc processing enzymes

There is strong evidence showing that OGT and OGA, the two O-GlcNAc processing enzymes that drive the cycling of GlcNAc group on and off proteins, are subjected to control by the circadian clock, but data on direct measurements of OGT and OGA enzyme activities over a daily cycle are still lacking to the best of our knowledge. Circadian transcriptomic and proteomic analyses showed that the *oga* mRNA and OGA protein oscillate in mouse livers and fly heads [27,71,115–119], while *ogt* mRNA but not OGT protein was observed to oscillate in mouse livers and fly heads [20,21,27,71,109,110,120–122]. We showed that in *Drosophila* fly bodies, the transcripts and encoded proteins of the two O-GlcNAc processing enzymes are modulated by both circadian and metabolic input [15]. This section is devoted to review potential molecular mechanisms that mediate metabolic and circadian regulation of OGT and OGA.

*ogt* mRNA and its encoded protein OGT are regulated by nutrient levels and nutrient-sensing pathways that are expected to be highly rhythmic over the day–night cycle. There are two nutrient-sensing pathways that are known to regulate OGT protein level, mTOR [52–54] and insulin signalling [55], and glucose itself [50,51,56] has also been shown to modulate *ogt* mRNA expression. Currently, it is unclear how expression of *ogt* mRNA and their encoded proteins are regulated by the circadian clock. The clock can potentially orchestrate rhythmic *ogt* mRNA expression by targeting rhythmically active transcription factors. Candidates include nuclear factor E2-related factor-2 (Nrf2) [77–80], E2F1 transcription factor [81,82] and hepatocyte nuclear factor 1 homologue A (HNF1A) [72,83,84], which are known to regulate *ogt* expression. With regard to OGT protein cycling, lysine-specific histone demethylase 1B (KDM1B or LSD2) and ubiquitin ligase E6AP have been shown to facilitate OGT ubiquitylation and degradation through their ubiquitin ligase activity [85,86]. Interestingly, *LSD2* and *E6AP* transcripts are both observed to oscillate in circadian transcriptome studies [71,72,82]. Finally, the clock protein REV-ERB*α* directly interacts with OGT and stabilizes OGT in different cellular compartments, as the cellular localization of REV-ERB*α* oscillates [87]. Whether and how these mechanisms contribute to circadian regulation of OGT levels will need to be explored in future studies. When compared with the regulation of *ogt* expression, much less is known about pathways that modulate *oga* expression. Given that E2F1 regulates *oga* expression in addition to *ogt* expression [81], it represents a transcription factor candidate [82] that can drive rhythmic *oga* expression.

At the post-transcriptional level, OGT enzymatic activity is regulated by multiple PTMs, which have been shown to respond to metabolic or circadian signals. Metabolic input has been shown to regulate OGT phosphorylation and thereby enzymatic activity through insulin signalling [21,57,58], as well as AMPK [59] and CAMKII [60,61] phosphorylation. Glycogen synthase kinase 3*β* (GSK3*β*), which happens to be an insulin signalling effector and a clock kinase, is shown to phosphorylate OGT at S3 or S4 to increase its enzymatic activity [21]. GSK3β-dependent phosphorylation of OGT can also change substrate selectivity [58]. Moreover, the circadian clock has been shown to rhythmically regulate the kinases and phosphatases that modify OGT, such as Checkpoint kinase 1 (ChK1) [88,89], Rho-associated coiled-coil forming protein kinase 2 (ROCK2) [71,72,90], myosin phosphatase target subunit 1 (MYPT1) [71,72,91,92]. Finally, O-GlcNAcylation of OGT S389 is shown to increase OGT nuclear localization [62]. As O-GlcNAcylation can integrate both metabolic and circadian signals [15], it will be interesting to explore whether OGT exhibits daily oscillation of subcellular localization. OGA can also be modified by phosphorylation and O-GlcNAcylation [123]. However, the functional role of these PTMs on OGA is less defined. Whether the phosphorylation and O-GlcNAcylation status of OGT and OGA is rhythmically regulated over a 24 h day–night cycle and how that modulates their activities needs future investigation.

To conclude the discussion on daily regulation of O-GlcNAc processing enzymes, it is important to point out that OGT and OGA impose reciprocal regulation against one another to maintain O-GlcNAc homeostasis. For example, as OGT protein level decreases, OGT forms a repressor complex with mSin3A and histone deacetylase1 (HDAC1) at the promoter of *oga* to inhibit its expression [64]. OGA can promote *ogt* expression by reducing O-GlcNAcylation of CCAAT/enhancer-binding protein*β* (C/EBP*β*) recruited to the promoter of *ogt* [63]. Notably, increasing O-GlcNAcylation level using an OGA inhibitor has been observed to elevate OGA protein while decreasing OGT protein level [93]. The reciprocal regulation of the two O-GlcNAc processing enzymes is expected to contribute to shaping daily rhythmicity of O-GlcNAcylation.

## 3. Crosstalk between O-GlcNAcylation and other post-translational modifications to regulate daily cellular physiology

**Figure 2.**
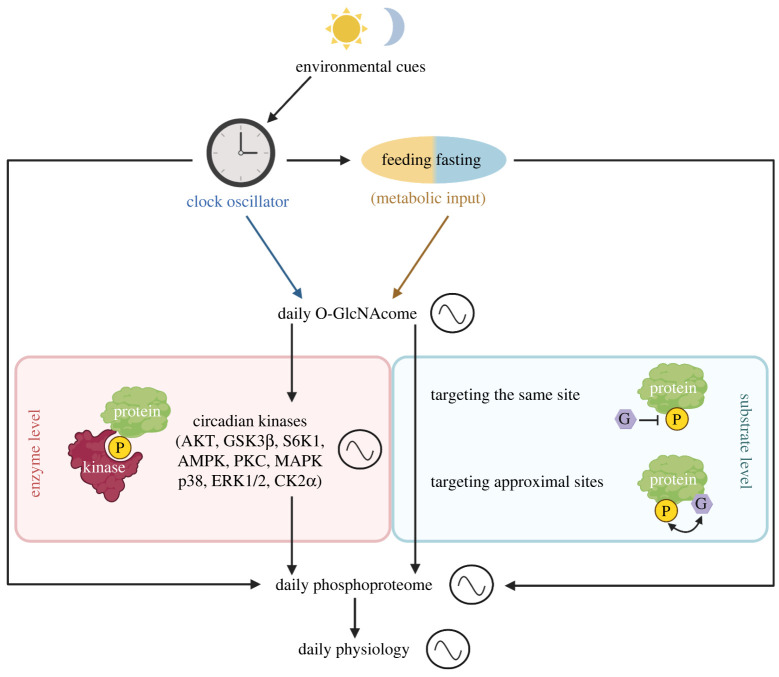
Daily rhythmicity of the O-GlcNAcome can integrate metabolic and circadian signals to modulate rhythmicity of the phosphoproteome. O-GlcNAcylation can modulate rhythmic activities of ‘circadian kinases’, which have been previously identified by analysing circadian/daily phosphoproteomic datasets [25,27]. We define this as enzyme level regulation. For substrate level regulation, O-GlcNAcylation can directly compete with phosphorylation by targeting the same residue on substrate proteins and/or modulate the protein conformation to promote or inhibit phosphorylation by targeting an approximal site [129–133]. Independently, phosphorylation is also sensitive to environmental and metabolic signals. In sum, interplay between daily phosphorylation and O-GlcNAcylation regulates time-of-day functions of cellular proteins and daily physiological rhythms. GSK3*β*, Glycogen synthase kinase3*β*; S6K1, ribosomal protein S6 kinase 1; AMPK, AMP-activated protein kinase; PKC, protein kinase C; MAPK p38, mitogen-activated protein kinase p38, ERK1/2, extracellular signal-regulated kinase1/2; CK2*α* casein kinase2*α*.

Different types of PTMs co-occur on proteins to regulate their functions in response to diverse physiological and environmental signals. O-GlcNAc modifications have been shown to exhibit crosstalk with other PTMs, such as phosphorylation (reviewed in [24]), acetylation [124–126] and ubiquitination [127,128]. The crosstalk between O-GlcNAcylation and phosphorylation has attracted the most attention, as both PTMs target serine and threonine residues. Given that circadian proteomics studies in recent years demonstrated that phospho-occupancy in many cellular proteins exhibit daily rhythmicity to regulate time-of-day protein functions [25–28], it is intriguing to explore how O-GlcNAcylation and phosphorylation (and possibly other PTMs) could work in conjunction to regulate daily rhythmicity in protein functions. Crosstalk between O-GlcNAcylation and other PTMs can present itself in two manners: (i) modify the function of writers and erasers (enzyme level), or (ii) modify the same sites or nearby sites on protein substrates to modulate the level of other PTMs (substrate level) (figure 2). With an emphasis on phosphorylation, we next review the mechanisms by which O-GlcNAcylation could shape daily rhythmicity in phosphoproteome to regulate biological rhythms.

### 3.1. Daily O-GlcNAcylation-phosphorylation crosstalk (O-P crosstalk) at the enzyme level

Since global O-GlcNAcylation level oscillates over a 24 h cycle [15] and O-GlcNAcylation has been shown to regulate the function of a myriad of kinases and phosphatases [24,134,135], time-of-day specific O-GlcNAcylation of kinases and phosphatases could represent an important mechanism to remodel daily rhythmicity in the phosphoproteome. In 2012, Dias *et al.* [136] systematically analysed O-GlcNAcylation of kinases using an *in vitro* OGT assay. They screened through 152 full-length human kinases and identified 42 O-GlcNAcylated kinases. More recently, Schwein & Woo [135] reviewed the O-GlcNAcomic datasets, and found more than 100 O-GlcNAcylated kinases, which covers all six major kinase families (AGC, CMGC, CAMK, STE, CK1 and TK/TKL) and some atypical protein kinases. Not surprisingly, a number of phosphatases are also found to be O-GlcNAcylated, including MYPT1, PPFIA2−4, PPP6R2, PTPN6, PTPN7, PTPRC, TNS2 and SIRPA [135].

Much progress has been made in characterizing the function of O-GlcNAcylation on many kinases. In table 1, we highlight the O-GlcNAcylated ‘circadian kinases', which we identified by analysing the circadian phosphoproteome [25,27]. Nevertheless, proteomic studies on how O-GlcNAc sites of certain kinases could modulate the phosphoproteome are still rather limited. Schwein *et al.* [146] recently used nanobody-OGT and nanobody-split OGA to specifically modify O-GlcNAc S347 on CK2*α* and analysed the phosphoproteome in HEK293 cells. They observed that increased CK2*α* O-GlcNAcylation promotes the phosphorylation of 39 proteins, enriched for chromatin modification, metabolism and ribosome, while decreasing the phosphorylation of 12 proteins. In conclusion, O-GlcNAcylation could regulate cellular physiology through modifying the kinome and thereby the phosphoproteome (figure 2). Future investigation is warranted to reveal the O-GlcNAcylation-kinome crosstalk under the framework of the 24 h day–night cycle.

**Table 1.** O-GlcNAcylation of kinases known to regulate circadian rhythm. GSK3*β*, glycogen synthase kinase3*β*; S6K1, ribosomal protein S6 kinase 1; AMPK, AMP-activated protein kinase; PKC, protein kinase C; MAPK p38, mitogen-activated protein kinase p38; ERK1/2, extracellular signal-regulated kinase1/2, CK2*α* casein kinase2*α*.

kinases	O-GlcNAc sites	function of O-GlcNAcylation	references
AKT	T308, S473 (characterized by mutagenesis)	inhibit AKT phosphorylation at T308 and S473, and inhibit AKT activity	[137,138]
GSK3*β*	n.a.	promote GSK3*β* phosphorylation at S9, and inhibit GSK3 activity	[137,139,140]
S6K1	S489 (characterized by mutagenesis)	inhibit S6K1 phosphorylation at S418 and T229, and inhibit S6K1 activity	[141]
AMPK	n.a.	inhibit AMPK*α* phosphorylation at T174, and inhibit AMPK activity	[59,142]
PKC*ζ*	T408, T410 (characterized by mutagenesis)	inhibit PKC*ζ* phosphorylation at T410, and inhibit PKC*ζ* activity	[143]
MAPK p38	n.a.	promote p38 phosphorylation, and activate p38 activity	[144]
ERK1/2	n.a.	promote ERK1/2 phosphorylation, and activate ERK1/2 activity	[144]
CK2*α*	S347 (validated by Edman sequencing)	inhibit CK2*α* phosphorylation at T344, reduce the interaction between CK2*α* and PIN1, promote CK2*α* degradation, and alter substrate selectivity	[145]

### 3.2. Daily O-P crosstalk at the substrate level

Since the discovery that O-GlcNAcylation and phosphorylation can modify the same amino acid residue on the same protein [147], crosstalk between O-GlcNAcylation and phosphorylation on substrates is recognized as an important mechanism for regulating protein function. Early study investigating O-P crosstalk used OGA inhibitors to elevate the global O-GlcNAcylation level and assayed the phosphoproteome in cell culture [129]. Out of the 711 phosphopeptides detected, 148 phosphopeptides increased and 280 decreased upon OGA inhibition. The phosphoproteins identified by Wang *et al.* [129] were enriched for cytoskeleton, cytoskeleton binding and RNA/DNA binding proteins. In addition to using inhibitors to globally alter protein O-GlcNAcylation status, other approaches have also been used to characterize physiologically relevant O-P crosstalks. Trinidad *et al.* [130] studied O-P crosstalks at murine synapses, as both OGT and OGA are enriched at synapses [148,149]. They sequentially enriched for O-GlcNAc and phosphopeptides and successfully detected O-GlcNAcylation and phosphorylation on the same peptides [130]. Among the 1750 O-GlcNAc sites and 16 500 phosphosites detected, 135 sites can be modified by both O-GlcNAcylation and phosphorylation. More recently, Fan *et al.* [131] developed a HILIC enrichment method to simultaneously enrich for O-GlcNAc and phosphopeptides. They assayed O-P crosstalk on 1115 RNA binding proteins (RBPs) and found that 213 RBPs (25%) can be both O-GlcNAcylated and phosphorylated. Taken together, direct competition between O-GlcNAcylation and phosphorylation may not be the dominant mechanism for O-P crosstalk, and O-GlcNAcylation and phosphorylation are more likely to regulate each other through modifying approximal sites (figure 2).

To elucidate the mechanisms for O-P crosstalk, researchers have started to analyse potential consensus sequence of O-P crosstalk. Although sites modified by both O-GlcNAcylation and phosphorylation occur at a relatively low frequency, Yao *et al.* [132] extracted three motifs (Pxx[S], Txxx[S] and [T]xxxxxxxxxP) that are overrepresented in S/T exhibiting O-P crosstalk at the same residue. For O-P crosstalk at approximal sites, Leney *et al.* [133] performed a systematic analysis using a MS-based *in vitro* kinetic assay and identified a motif with four amino acids: N-[S/T]P(V/A/T)[S/T]-C. Phosphorylation occurs at the N terminal S/T and O-GlcNAcylation modifies the C terminal S/T, and the two PTMs tend to reciprocally inhibit one other [133]. Data mining in PhosphoSite Plus showed that 1048 proteins could be regulated by this potential mechanism, and previous studies support that O-P crosstalk on proteins such as eukaryotic initiation factor 4 (eIF4) and Sin3A could be mediated by this motif [133,150,151]. Kinase prediction shows that extracellular signal-regulated kinase1 (ERK1), ERK2, CK1 and GSK3*β* are likely to modify the motifs mentioned above [132,133]. Interestingly, these kinases overlap with ‘circadian kinases' that can rhythmically phosphorylate proteins over a 24 h cycle [25,27], suggesting that daily cycling of O-GlcNAcylation could potentially regulate daily rhythmicity in the phosphoproteome at the substrate level (figure 2). Nevertheless, this hypothesis needs to be tested with the advances of O-P peptide enrichment methods and MS proteomics.

## 4. O-GlcNAcylation is an important mechanism that regulates daily cellular physiology

**Figure 3.**
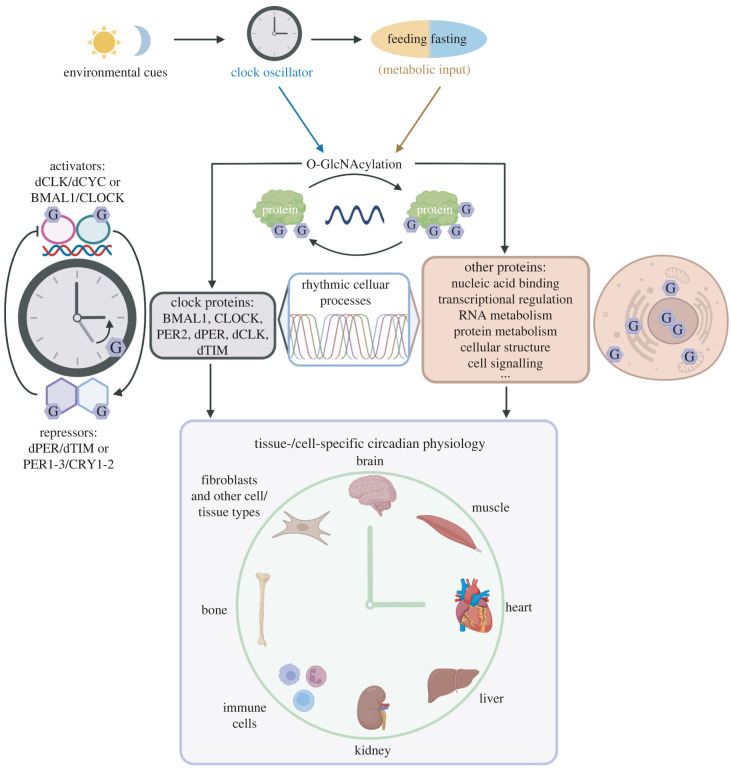
O-GlcNAcylation regulates daily biological rhythms from cellular to organismal level. O-GlcNAcylation rhythmically modifies circadian clock proteins, key components of the molecular oscillator [21–23]. Global increase in cellular O-GlcNAcylation slows down the pace of circadian clocks, which in turn alters timing of rhythmic cellular processes [20–22]. In addition to clock proteins, thousands of other cellular proteins are also O-GlcNAcylated. O-GlcNAcylation is directly involved in regulating basic cellular functions, such as transcriptional regulation, RNA metabolism, translation, protein metabolism [16–19]. Furthermore, O-GlcNAcylation can also modify activities of organ-, tissue- or cell-specific processes [153–173]. In summary, rhythmic O-GlcNAcylation ranging from subcellular to organ levels manifest into robust daily biological rhythms at the organismal level. dCLK, *Drosophila* CLOCK; dCYC *Drosophila* CYCLE; BMAL1, brain and muscle Arnt-like protein-1; CLOCK, circadian locomotor output cycles kaput; dPER, *Drosophila* PERIOD; dTIM, *Drosophila* TIMELESS; PER1-3, PERIOD1-3; CRY1-2, CRYPTOCHROME1-2.

A large body of work contributed to our understanding of diverse mechanisms by which metabolic input interacts with the endogenous circadian clock to regulate daily biological rhythms (reviewed in [11,13,152]). Our recent study highlights protein O-GlcNAcylation as a key post-translational mechanism that integrates metabolic and circadian signals to regulate rhythmic physiology [15]. At the molecular level, there are two ways O-GlcNAcylation can regulate daily rhythms of cellular physiology: (i) O-GlcNAcylation can modulate core clock proteins and the pace of the molecular clock, which in turn alters rhythmicity of diverse cellular processes; (ii) O-GlcNAcylation can rhythmically modify cellular proteins outside of the molecular oscillator to regulate their time-of-day specific functions (figure 3). In this section, we review the impact of O-GlcNAcylation on clock protein functions and outline other rhythmic cellular processes that can be modified by O-GlcNAcylation beyond the core clock.

### 4.1. Regulation of clock proteins within the core molecular oscillator by O-GlcNAcylation

The molecular oscillator of the animal circadian timing system relies on transcription-translation feedback mechanisms to maintain approximately 24 h biological rhythms (reviewed in [1,2]) (figure 3). Brain and muscle Arnt-like protein-1 (BMAL1) and circadian locomotor output cycles kaput (CLOCK) are the key transcriptional activators of the mammalian clock (*Drosophila* homologues are dCYCLE and dCLOCK (dCLK)), and as heterodimers, they drive the expression of thousands of clock-controlled genes including genes that encode their own transcriptional repressors, PERIOD1-3 (PER1-3) and CRYPTOCHROME1-2 (CRY1-2) (dPER and dTIMELESS (dTIM) in *Drosophila*) (figure 3). The molecular oscillator is critical for generating daily rhythmicity of gene expression that manifest into a range of rhythmic biological processes (reviewed in [1–10]).

PTMs, especially phosphorylation, have been established as essential mechanisms for maintaining the pace of the molecular oscillator [174–176]. Phosphorylation of the clock protein dPER was first characterized by Edery *et al.* [177] and subsequent studies continue to highlight diverse properties of clock proteins that are regulated by phosphorylation (reviewed in [178,179]). O-GlcNAcylation was first introduced as a mechanism to regulate the molecular clock and circadian rhythms by Kim *et al.* [20], Kaasik *et al.* [21] and Li *et al.* [22]. These pioneering studies showed that in both fly and mammalian models, increasing global O-GlcNAcylation slows down the pace of the clock and results in period lengthening of behavioural rhythms, while reducing O-GlcNAcylation has the opposite effect. Furthermore, these studies and subsequent studies [23,180] showed that many clock proteins, including dCLK, dPER, dTIM, BMAL1, CLOCK, are O-GlcNAcylated and period-altering effects are mediated by disrupting clock protein O-GlcNAcylation.

Interestingly but perhaps not surprisingly, some clock proteins even display daily rhythms of O-GlcNAcylation that are sensitive to feeding and nutrient input [21–23]. In *Drosophila*, O-GlcNAcylation of dPER promotes its stability and inhibits nuclear entry [20]. Transcriptional reporter assays in *Drosophila* S2 tissue culture showed that manipulating O-GlcNAcylation levels by overexpressing OGT or OGA also changes dPER and dCLK transcriptional activities [21]. To begin to dissect site-specific O-GlcNAc regulation, our group mapped dPER and dTIM O-GlcNAc sites in fly heads using MS proteomics [23,180]. We found that O-GlcNAcylation of dPER S942 inhibits the interaction of dPER and dCLK and reduces dPER repressor activity [23]. The function of O-GlcNAcylation on dTIM however remains to be determined. In mammalian clock, O-GlcNAcylation is shown to stabilize BMAL1 and CLOCK by inhibiting their ubiquitination [22]. In HEK293 cells, O-GlcNAcylation and phosphorylation compete at PER2 S662, and O-GlcNAcylation of S662 increases PER2 repressor activity [21]. In summary, O-GlcNAcylation is highly involved in the regulation of molecular oscillators (figure 3). Future site-specific characterization of clock proteins is needed to further understand the mechanisms by which metabolic input regulates molecular oscillators and biological rhythms through O-GlcNAcylation.

### 4.2. Regulation of rhythmic cellular processes beyond the molecular oscillator by O-GlcNAcylation

O-GlcNAcylation not only occurs on clock proteins, but also regulates the function of a large part of the proteome (figure 3). By cataloging results from over 1700 articles, O-GlcNAcome Database listed 7789 human O-GlcNAc proteins and 3503 mouse O-GlcNAc proteins [18]. Meta analysis on the human O-GlcNAcylated proteins from Wulff-Fuentes *et al.* [18] and combing through published O-GlcNAcomic papers [153–173] suggest that O-GlcNAc proteins are heavily involved in nucleic acid binding/transcriptional regulation/RNA metabolism, metabolism of proteins, cellular structure and cell signalling (figure 3). Many elegant reviews have summarized published functional investigations of O-GlcNAcylation on cellular proteins (e.g. [16,17,19,181,182]). To date, although circadian/daily rhythm of the O-GlcNAcome has yet to be conducted, our study showing robust daily rhythmicity in global protein O-GlcNAcylation in *Drosophila* tissues suggests that diverse cellular processes and molecular pathways could potentially be rhythmically regulated by daily cycling O-GlcNAcylation that is sensitive to metabolic input [15].

In table 2, we summarize published efforts to identify O-GlcNAcylated proteins in different cell types or tissues, which could provide insights into tissue- or cell-specific daily O-GlcNAc regulation on biological rhythms. In particular, we highlight pathways with O-GlcNAcylated factors that are critical for performing tissue- or cell-specific functions. We excluded O-GlcNAcomic studies conducted using cancer cell lines, as cellular O-GlcNAc status is known to be altered in cancer cells compared to cells under physiological conditions [183–185]. Finally, we also excluded studies conducted using whole organisms, such as *D. melanogaster* and *Caenorhabditis elegans* [186–188], since our focus in table 2 is on tissue-specific characterization.

**Table 2.** O-GlcNAcomic studies in animal tissues and cell lines.

tissue or cell type	organism	number of O-GlcNAc proteins	number of O-GlcNAc sites	tissue- or cell-specific function of O-GlcNAcylation	references
**nervous system**					
forebrain	rat	25	n.a.	cellular communication/signal transduction; intracellular transport	[153,154]
hippocampus	mouse	14	n.a.	neuronal structure; glucose metabolism	[155]
cerebral cortical tissue	mouse	274	n.a.	neurogenesis; synaptic transmission; learning and memory; cytoskeleton	[156]
cortex	mouse	278	n.a.	synaptic trafficking; notch/Wnt signalling; circadian clock proteins	[157]
brain	rat	30	n.a.	signal transduction; cytoskeleton and vesicle trafficking	[158]
brain	human	530	1094	receptor signalling; substrate-adhesion dependent cell spreading; cell projection assembly	[159]
**muscular system**					
gastrocnemius muscle	rat	14	n.a.	glycolytic pathway and energetic metabolism; contractile protein	[160]
C2C12 myotubes	mouse	342	n.a.	cytoskeleton and chaperones; transporter and binding proteins; cell adhesion molecules	[161]
right ventricle	rat	500	n.a.	oxidation–reduction process; intracellular transport; metabolism; cellular respiration and energy	[162]
**excretory system**					
embryonic kidney cells (HEK293)	human	1500	180	cell death; molecular transport; cellular assembly and organization; cell cycle, growth and proliferation; cell morphology; PTM	[163]
embryonic kidney cells (HEK293)	human	75	n.a.	cell-cell adhesion; cell cycle; molecular transport; Purine ribonucleoside monophosphate biosynthetic process; cellular response to heat; viral process	[164]
embryonic kidney cells (HEK293)	human	215	n.a.	Metabolism; Signal transduction; Translation; Transport	[165]
urine	human	457	n.a.	organelle organization; cell cycle; cellular localization; heterocycle metabolic processes; DNA repair; cellular response to stress; developmental processes; transport	[166]
**immune system**					
T cell	mouse	116	n.a.	metabolic process; cellular component organization/biogenesis; DNA packing	[167]
T cell	human	133	n.a.	nucleotide, nucleic acid transport	[168]
T cell	human	1045	n.a.	viral process; cell-cell adhesion; cell cycle; cellular transport; protein sumoylation	[169]
embryonic macrophage-like cells (S2 cells)	fruit fly	51	n.a.	metabolism; stress response; cell cycle	[170]
**other tissues or cell types**					
liver	rat	68	n.a.	metabolism; transport; signal transduction	[165]
osteoblasts (MC3T3E1)	mouse	20	n.a.	post-translational regulation; systemic nutrient homeostasis	[171]
placental trophoblasts (BeWo)	human	829	n.a.	translational initiation; viral transcription; SRP-dependent co-translational protein targeting to membrane	[172]
fibroblasts (NIH3T3)	mouse	374	n.a.	metabolism; intracellular transport	[173]

In addition to tissue- or cell-specific studies, there are other in depth O-GlcNAcomic studies at the organelle level, such as mitochondria from rat heart [189,190] and rat liver [191], cardiac myofilament from rat [192], synapses from mouse brain [130,193,194], ribosome from rat liver [195] and nuclei from mouse embryonic stem cells [196]. Additionally, comparative O-GlcNAcomic studies have been carried out in cell culture systems to investigate the role of O-GlcNAcylation under different conditions [197–199] or during the progression of cell cycles [200,201]. However, comparative O-GlcNAcomic studies over different time points of a 24 h day and in different organs/tissues *in vivo* are warranted to reveal the role of O-GlcNAcylation in regulating daily rhythmicity in organ- and tissue-specific physiology and potential differential regulation by metabolic and circadian signals.

## 5. Conclusion

Significant progress has been made to elucidate the regulation of metabolic signals on daily rhythms in physiology and behaviour. Metabolic input regulates gene expression at specific times of day through nutrient-sensing pathways influenced by feeding–fasting cycles. This is accomplished through functional modification of core clock proteins and/or epigenetic regulation of the genomic landscape. Our recent study showed that O-GlcNAcylation is also an important mechanism that integrates metabolic and circadian signals to regulate the daily biological rhythms [15]. In this review, we summarize published and potential mechanisms by which metabolic and circadian signals can shape daily O-GlcNAc oscillation, discuss crosstalk between O-GlcNAcylation and the phosphoproteome to regulate rhythmic protein functions, and highlight cellular pathways that may be regulated by oscillating O-GlcNAcylation in different tissues or cell types. As time-restricted feeding/eating is emerging as a non-invasive therapeutic strategy to alleviate metabolic syndromes [30,35,202,203], our review provides mechanistic insight into the significance of properly aligning our eating time with biological rhythm. Since we showed in *Drosophila* that food consumption at unnatural feeding time of the day–night cycle can dampen the oscillation of protein O-GlcNAcylation [15], it is likely that rhythmic functions of O-GlcNAc proteins/pathways would be disrupted with mistimed eating. This may contribute to deleterious effects of mistimed eating and high-fat diet, which has been shown to impair feeding–fasting rhythms and rhythmic metabolic input [204,205].

It is important to note that different organs or tissues are likely differentially regulated by metabolic input versus circadian input. For example, despite that the blood–brain barrier is expected to render brain tissues less sensitive to daily oscillation of metabolites, O-GlcNAcylation has been detected in brain tissues and shown to oscillate on clock proteins in fly heads [20,21,23]. Our understanding of the similarities and divergence among O-GlcNAcomes in multiple organs or tissues and how organisms coordinate organ/tissue-specific O-GlcNAcomic rhythms to properly maintain time-of-day physiology at the organismal level will improve with continued development of comparative O-GlcNAcomic methods [198,201].

In this review, we largely focused on O-GlcNAcylation as an intracellular mechanism that underlies metabolic regulation of daily biological rhythms. We briefly mentioned a few intercellular signals, such as insulin, EGF, TGF*β*, which could contribute to regulation of protein O-GlcNAcylation. However, there are many other intercellular signals, including neuronal signals, hormones (melatonin, adrenal cortex hormones, thyroid hormones etc.) and gut microbiota, which can relay time-of-day specific metabolic signals to influence cellular protein functions. How O-GlcNAcylation responds to these intercellular signals is unknown and beyond the scope of this review.

Finally, it is important to note that O-GlcNAcylation is only one of many nutrient-sensitive PTMs. Feeding–fasting cycles likely regulate daily cellular physiology through other metabolite-driven PTMs. Figlia *et al.* [206] reviewed over 20 different types of PTM using metabolites, such as lipids, amino acids, Coenzyme-A, acetate, malonate and lactate. Future studies are warranted to determine whether these nutrient-sensitive PTMs are also involved in regulation of daily biological rhythms. Recently, Bludau *et al.* [207] developed an exciting tool to predict protein structure with PTMs. In combination with site-specific information of these metabolite-driven PTMs, the metabolic regulation of protein functions could be computationally predicted, which could provide a more comprehensive view on the metabolic effect on daily biological rhythms.

## Data Availability

This article has no additional data.

## References

[1] Cox KH, Takahashi JS. 2019 Circadian clock genes and the transcriptional architecture of the clock mechanism. J. Mol. Endocrinol. **63**, R93-R102. (10.1530/JME-19-0153) 31557726 PMC6872945

[2] Patke A, Young MW, Axelrod S. 2020 Molecular mechanisms and physiological importance of circadian rhythms. Nat. Rev. Mol. Cell Biol. **21**, 67-84. (10.1038/s41580-019-0179-2) 31768006

[3] Deota S, Panda S. 2021 New horizons: circadian control of metabolism offers novel insight into the cause and treatment of metabolic diseases. J. Clin. Endocrinol. Metab. **106**, e1488-e1493. (10.1210/clinem/dgaa691) 32984881 PMC7947830

[4] Ono D. 2022 Neural circuits in the central circadian clock and their regulation of sleep and wakefulness in mammals. Neurosci. Res. **182**, 1-6. (10.1016/j.neures.2022.05.005) 35597406

[5] Yildirim E, Curtis R, Hwangbo D. 2022 Roles of peripheral clocks: lessons from the fly. FEBS Lett. **596**, 263-293. (10.1002/1873-3468.14251) 34862983 PMC8844272

[6] Peng F, Li X, Xiao F, Zhao R, Sun Z. 2022 Circadian clock, diurnal glucose metabolic rhythm, and dawn phenomenon. Trends Neurosci. **45**, 471-482. (10.1016/j.tins.2022.03.010) 35466006 PMC9117496

[7] Yao Y, Silver R. 2022 Mutual shaping of circadian body-wide synchronization by the suprachiasmatic nucleus and circulating steroids. Front. Behav. Neurosci. **16**, 877256. (10.3389/fnbeh.2022.877256) 35722187 PMC9200072

[8] Wang C, Lutes LK, Barnoud C, Scheiermann C. 2022 The circadian immune system. Sci. Immunol. **7**, eabm2465. (10.1126/sciimmunol.abm2465) 35658012

[9] Tabuchi M, Coates KE, Bautista OB, Zukowski LH. 2021 Light/clock influences membrane potential dynamics to regulate sleep states. Front. Neurol. **12**, 625369. (10.3389/fneur.2021.625369) 33854471 PMC8039321

[10] Cuddapah VA, Zhang SL, Sehgal A. 2019 Regulation of the blood–brain barrier by circadian rhythms and sleep. Trends Neurosci. **42**, 500-510. (10.1016/j.tins.2019.05.001) 31253251 PMC6602072

[11] Panda S. 2016 Circadian physiology of metabolism. Science **354**, 1008-1015. (10.1126/science.aah4967) 27885007 PMC7261592

[12] Mauvoisin D et al. 2017 Circadian and feeding rhythms orchestrate the diurnal liver acetylome. Cell Rep. **20**, 1729-1743. (10.1016/j.celrep.2017.07.065) 28813682 PMC5568034

[13] Sato T, Sassone-Corsi P. 2022 Nutrition, metabolism, and epigenetics: pathways of circadian reprogramming. EMBO Rep. **23**, e52412. (10.15252/embr.202152412) 35412705 PMC9066069

[14] Shu XE, Swanda RV, Qian S-B. 2020 Nutrient control of mRNA Translation. Annu. Rev. Nutr. **40**, 51-75. (10.1146/annurev-nutr-120919-041411) 32631146

[15] Liu X, Blaženović I, Contreras AJ, Pham TM, Tabuloc CA, Li YH, Ji J, Fiehn O, Chiu JC. 2021 Hexosamine biosynthetic pathway and O-GlcNAc-processing enzymes regulate daily rhythms in protein O-GlcNAcylation. Nat. Commun. **12**, 4173. (10.1038/s41467-021-24301-7) 34234137 PMC8263742

[16] Hart GW. 2019 Nutrient regulation of signaling and transcription. J. Biol. Chem. **294**, 2211-2231. (10.1074/jbc.AW119.003226) 30626734 PMC6378989

[17] Chatham JC, Zhang J, Wende AR. 2021 Role of *O*-linked *N*-acetylglucosamine protein modification in cellular (patho)physiology. Physiol. Rev. **101**, 427-493. (10.1152/physrev.00043.2019) 32730113 PMC8428922

[18] Wulff-Fuentes E, Berendt RR, Massman L, Danner L, Malard F, Vora J, Kahsay R, Olivier-Van Stichelen S. 2021 The human O-GlcNAcome database and meta-analysis. Sci. Data **8**, 25. (10.1038/s41597-021-00810-4) 33479245 PMC7820439

[19] Ma J, Hou C, Wu C. In press. Demystifying the O-GlcNAc code: a systems view. Chem. Rev. 10.1021/acs.chemrev.1c01006) 35302357

[20] Kim EY, Jeong EH, Park S, Jeong H-J, Edery I, Cho JW. 2012 A role for *O*-GlcNAcylation in setting circadian clock speed. Genes Dev. **26**, 490-502. (10.1101/gad.182378.111) 22327476 PMC3305986

[21] Kaasik K et al. 2013 Glucose sensor O-GlcNAcylation coordinates with phosphorylation to regulate circadian clock. Cell Metab. **17**, 291-302. (10.1016/j.cmet.2012.12.017) 23395175 PMC3597447

[22] Li M-D, Ruan H-B, Hughes ME, Lee J-S, Singh JP, Jones SP, Nitabach MN, Yang X. 2013 O-GlcNAc signaling entrains the circadian clock by inhibiting BMAL1/CLOCK ubiquitination. Cell Metab. **17**, 303-310. (10.1016/j.cmet.2012.12.015) 23395176 PMC3647362

[23] Li YH, Liu X, Vanselow JT, Zheng H, Schlosser A, Chiu JC. 2019 O-GlcNAcylation of PERIOD regulates its interaction with CLOCK and timing of circadian transcriptional repression. PLoS Genet. **15**, e1007953. (10.1371/journal.pgen.1007953) 30703153 PMC6372208

[24] Laarse SAM, Leney AC, Heck AJR. 2018 Crosstalk between phosphorylation and O-GlcNAcylation: friend or foe. FEBS J. **285**, 3152-3167. (10.1111/febs.14491) 29717537

[25] Robles MS, Humphrey SJ, Mann M. 2017 Phosphorylation is a central mechanism for circadian control of metabolism and physiology. Cell Metab. **25**, 118-127. (10.1016/j.cmet.2016.10.004) 27818261

[26] Horta MAC et al. 2019 Broad substrate-specific phosphorylation events are associated with the initial stage of plant cell wall recognition in *Neurospora crassa*. Front. Microbiol. **10**, 2317. (10.3389/fmicb.2019.02317) 31736884 PMC6838226

[27] Wang C et al. 2020 Integrated omics in *Drosophila* uncover a circadian kinome. Nat. Commun. **11**, 2710. (10.1038/s41467-020-16514-z) 32483184 PMC7264355

[28] Krahmer J, Hindle M, Perby LK, Mogensen HK, Nielsen TH, Halliday KJ, van Ooijen G, Le Bihan T, Millar AJ. 2022 The circadian clock gene circuit controls protein and phosphoprotein rhythms in *Arabidopsis thaliana*. Mol. Cell. Proteomics **21**, 100172. (10.1016/j.mcpro.2021.100172) 34740825 PMC8733343

[29] Acosta-Rodríguez V, Rijo-Ferreira F, Izumo M, Xu P, Wight-Carter M, Green CB, Takahashi JS. 2022 Circadian alignment of early onset caloric restriction promotes longevity in male C57BL/6 J mice. Science **376**, 1192-1202. (10.1126/science.abk0297) 35511946 PMC9262309

[30] Chaix A, Manoogian ENC, Melkani GC, Panda S. 2019 Time-restricted eating to prevent and manage chronic metabolic diseases. Annu. Rev. Nutr. **39**, 291-315. (10.1146/annurev-nutr-082018-124320) 31180809 PMC6703924

[31] Chaix A, Lin T, Le HD, Chang MW, Panda S. 2019 Time-restricted feeding prevents obesity and metabolic syndrome in mice lacking a circadian clock. Cell Metab. **29**, 303-319.e4. (10.1016/j.cmet.2018.08.004) 30174302 PMC7751278

[32] Chaix A, Zarrinpar A, Miu P, Panda S. 2014 Time-restricted feeding is a preventative and therapeutic intervention against diverse nutritional challenges. Cell Metab. **20**, 991-1005. (10.1016/j.cmet.2014.11.001) 25470547 PMC4255155

[33] Chaix A, Deota S, Bhardwaj R, Lin T, Panda S. 2021 Sex- and age-dependent outcomes of 9-hour time-restricted feeding of a Western high-fat high-sucrose diet in C57BL/6 J mice. Cell Rep. **36**, 109543. (10.1016/j.celrep.2021.109543) 34407415 PMC8500107

[34] Gill S, Le HD, Melkani GC, Panda S. 2015 Time-restricted feeding attenuates age-related cardiac decline in *Drosophila*. Science **347**, 1265-1269. (10.1126/science.1256682) 25766238 PMC4578815

[35] Hatori M et al. 2012 Time-restricted feeding without reducing caloric intake prevents metabolic diseases in mice fed a high-fat diet. Cell Metab. **15**, 848-860. (10.1016/j.cmet.2012.04.019) 22608008 PMC3491655

[36] Very N, Vercoutter-Edouart A-S, Lefebvre T, Hardivillé S, El Yazidi-Belkoura I. 2018 Cross-dysregulation of O-GlcNAcylation and PI3 K/AKT/mTOR axis in human chronic diseases. Front. Endocrinol. **9**, 602. (10.3389/fendo.2018.00602) PMC6189293 30356686

[37] Weigert C, Klopfer K, Kausch C, Brodbeck K, Stumvoll M, Häring HU, Schleicher ED. 2003 Palmitate-induced activation of the hexosamine pathway in human myotubes. Diabetes **52**, 650-656. (10.2337/diabetes.52.3.650) 12606504

[38] Chaveroux C et al. 2016 Nutrient shortage triggers the hexosamine biosynthetic pathway via the GCN2-ATF4 signalling pathway. Sci. Rep. **6**, 27278. (10.1038/srep27278) 27255611 PMC4891703

[39] Paterson AJ, Kudlow JE. 1995 Regulation of glutamine:fructose-6-phosphate amidotransferase gene transcription by epidermal growth factor and glucose. Endocrinology **136**, 2809-2816. (10.1210/endo.136.7.7789306) 7789306

[40] Dai W, Dierschke SK, Toro AL, Dennis MD. 2018 Consumption of a high fat diet promotes protein O-GlcNAcylation in mouse retina via NR4A1-dependent GFAT2 expression. Biochim. Biophys. Acta **1864**, 3568-3576. (10.1016/j.bbadis.2018.09.006) PMC6239931 30254013

[41] Liu B, Huang Z-B, Chen X, See Y-X, Chen Z-K, Yao H-K. 2019 Mammalian target of rapamycin 2 (MTOR2) and C-MYC modulate glucosamine-6-phosphate synthesis in glioblastoma (GBM) cells through glutamine:fructose-6-phosphate aminotransferase 1 (GFAT1). Cell. Mol. Neurobiol. **39**, 415-434. (10.1007/s10571-019-00659-7) 30771196 PMC11469801

[42] Zheng H, Huang J, Zhang M, Zhao H-J, Chen P, Zeng Z-H. 2022 miR-27b-3p improved high glucose-induced spermatogenic cell damage via regulating Gfpt1/HBP signaling. Eur. Surg. Res. **63**, 64-76. (10.1159/000518960) 34986481

[43] Moloughney JG et al. 2016 mTORC2 responds to glutamine catabolite levels to modulate the hexosamine biosynthesis enzyme GFAT1. Mol. Cell **63**, 811-826. (10.1016/j.molcel.2016.07.015) 27570073 PMC5006067

[44] Eguchi S, Oshiro N, Miyamoto T, Yoshino K, Okamoto S, Ono T, Kikkawa U, Yonezawa K. 2009 AMP-activated protein kinase phosphorylates glutamine:fructose-6-phosphate amidotransferase 1 at Ser243 to modulate its enzymatic activity. Genes Cells **14**, 179-189. (10.1111/j.1365-2443.2008.01260.x) 19170765

[45] Zibrova D et al. 2017 GFAT1 phosphorylation by AMPK promotes VEGF-induced angiogenesis. Biochem. J. **474**, 983-1001. (10.1042/BCJ20160980) 28008135

[46] Moloughney JG et al. 2018 mTORC2 modulates the amplitude and duration of GFAT1 Ser-243 phosphorylation to maintain flux through the hexosamine pathway during starvation. J. Biol. Chem. **293**, 16 464-16 478. (10.1074/jbc.RA118.003991) PMC6200946 30201609

[47] Graack HR, Cinque U, Kress H. 2001 Functional regulation of glutamine:fructose-6-phosphate aminotransferase 1 (GFAT1) of *Drosophila melanogaster* in a UDP-N-acetylglucosamine and cAMP-dependent manner. Biochem. J. **360**, 401-412. (10.1042/0264-6021:3600401) 11716769 PMC1222241

[48] Broschat KO, Gorka C, Page JD, Martin-Berger CL, Davies MS, Huang H, Gulve EA, Salsgiver WJ, Kasten TP. 2002 Kinetic characterization of human glutamine-fructose-6-phosphate amidotransferase I. J. Biol. Chem. **277**, 14 764-14 770. (10.1074/jbc.M201056200) 11842094

[49] Ruegenberg S, Horn M, Pichlo C, Allmeroth K, Baumann U, Denzel MS. 2020 Loss of GFAT-1 feedback regulation activates the hexosamine pathway that modulates protein homeostasis. Nat. Commun. **11**, 687. (10.1038/s41467-020-14524-5) 32019926 PMC7000685

[50] Taylor RP, Geisler TS, Chambers JH, McClain DA. 2009 Up-regulation of O-GlcNAc transferase with glucose deprivation in HepG2 cells is mediated by decreased hexosamine pathway flux. J. Biol. Chem. **284**, 3425-3432. (10.1074/jbc.M803198200) 19073609 PMC2635029

[51] Zou L, Zhu-Mauldin X, Marchase RB, Paterson AJ, Liu J, Yang Q, Chatham JC. 2012 Glucose deprivation-induced increase in protein O-GlcNAcylation in cardiomyocytes is calcium-dependent. J. Biol. Chem. **287**, 34 419-34 431. (10.1074/jbc.M112.393207) PMC3464547 22908225

[52] Zhang F, Snead CM, Catravas JD. 2012 Hsp90 regulates *O*-linked β-*N*-acetylglucosamine transferase: a novel mechanism of modulation of protein *O*-linked β-*N*-acetylglucosamine modification in endothelial cells. Am. J. Physiol.-Cell Physiol. **302**, C1786-C1796. (10.1152/ajpcell.00004.2012) 22496241 PMC3378080

[53] Park S, Pak J, Jang I, Cho J. 2014 Inhibition of mTOR affects protein stability of OGT. Biochem. Biophys. Res. Commun. **453**, 208-212. (10.1016/j.bbrc.2014.05.047) 24858682

[54] Sodi VL, Khaku S, Krutilina R, Schwab LP, Vocadlo DJ, Seagroves TN, Reginato MJ. 2015 mTOR/MYC axis regulates O-GlcNAc transferase expression and O-GlcNAcylation in breast cancer. Mol. Cancer Res. **13**, 923-933. (10.1158/1541-7786.MCR-14-0536) 25636967 PMC4433402

[55] Perez-Cervera Y, Dehennaut V, Gil MA, Guedri K, Mata CJS, Stichelen SO, Michalski J, Foulquier F, Lefebvre T. 2013 Insulin signaling controls the expression of *O*-GlcNAc transferase and its interaction with lipid microdomains. FASEB J. **27**, 3478-3486. (10.1096/fj.12-217984) 23689613

[56] Lo W-Y, Yang W-K, Peng C-T, Pai W-Y, Wang H-J. 2018 MicroRNA-200a/200b modulate high glucose-induced endothelial inflammation by targeting O-linked N-acetylglucosamine transferase expression. Front. Physiol. **9**, 355. (10.3389/fphys.2018.00355) 29720943 PMC5915961

[57] Whelan SA, Lane MD, Hart GW. 2008 Regulation of the O-linked β-N-acetylglucosamine transferase by insulin signaling. J. Biol. Chem. **283**, 21 411-21 417. (10.1074/jbc.M800677200) PMC2490780 18519567

[58] Wang Z, Pandey A, Hart GW. 2007 Dynamic interplay between O-Linked N-acetylglucosaminylation and glycogen synthase kinase-3-dependent phosphorylation. Mol. Cell. Proteomics **6**, 1365-1379. (10.1074/mcp.M600453-MCP200) 17507370

[59] Bullen JW, Balsbaugh JL, Chanda D, Shabanowitz J, Hunt DF, Neumann D, Hart GW. 2014 Cross-talk between two essential nutrient-sensitive enzymes. J. Biol. Chem. **289**, 10 592-10 606. (10.1074/jbc.M113.523068) PMC4036179 24563466

[60] Huttlin EL et al. 2010 A tissue-specific atlas of mouse protein phosphorylation and expression. Cell **143**, 1174-1189. (10.1016/j.cell.2010.12.001) 21183079 PMC3035969

[61] Ruan H-B et al. 2017 Calcium-dependent O-GlcNAc signaling drives liver autophagy in adaptation to starvation. Genes Dev. **31**, 1655-1665. (10.1101/gad.305441.117) 28903979 PMC5647936

[62] Seo HG, Kim HB, Kang MJ, Ryum JH, Yi EC, Cho JW. 2016 Identification of the nuclear localisation signal of O-GlcNAc transferase and its nuclear import regulation. Sci. Rep. **6**, 34614. (10.1038/srep34614) 27713473 PMC5054401

[63] Qian K et al. 2018 Transcriptional regulation of O-GlcNAc homeostasis is disrupted in pancreatic cancer. J. Biol. Chem. **293**, 13 989-14 000. (10.1074/jbc.RA118.004709) PMC6130940 30037904

[64] Vaidyanathan K et al. 2017 Identification and characterization of a missense mutation in the O-linked β-N-acetylglucosamine (O-GlcNAc) transferase gene that segregates with X-linked intellectual disability. J. Biol. Chem. **292**, 8948-8963. (10.1074/jbc.M116.771030) 28302723 PMC5448127

[65] Tuomela T, Viinikka L, Perheentupa J. 1990 Epidermal growth factor in mice: changes during circadian and female reproductive cycles. Acta Endocrinol. (Copenh.) **123**, 643-648. (10.1530/acta.0.1230643) 2284889

[66] Haus E, Haus E, Dumitriu L, Nicolau GY, Bologa S, Sackett-Lundeen L. 2001 Circadian rhythms of basic fibroblast growth factor (bFGF), epidermal growth factor (EGF), insulin-like growth factor-1 (IGF-1), insulin-like growth factor binding protein-3 (IGFBP-3), cortisol, and melatonin in women with breast cancer. Chronobiol. Int. **18**, 709-727. (10.1081/CBI-100106083) 11587092

[67] Lucena MC et al. 2016 Epithelial mesenchymal transition induces aberrant glycosylation through hexosamine biosynthetic pathway activation. J. Biol. Chem. **291**, 12 917-12 929. (10.1074/jbc.M116.729236) PMC4933211 27129262

[68] Vaghefi SSE, Mousavi F, Khaksari M, Asadikaram G, Soltani Z. 2021 Sex-related changes in circadian rhythm of inflammatory and oxidative stress markers in CKD. Iran J. Kidney Dis. **15**, 351-363. (10.52547/ijkd.6242) 34582370

[69] Yang H, Yang L-T, Liu J, Tang S, Zhao X, Wang Q, Zhang S, Pan W, Yang P-C. 2018 Circadian protein CLK suppresses transforming growth factor-β expression in peripheral B cells of nurses with day-night shift rotation. Am. J. Transl. Res. **10**, 4331-4337. 30662675 PMC6325493

[70] Yamazaki K, Mizui Y, Oki T, Okada M, Tanaka I. 2000 Cloning and characterization of mouse glutamine:fructose-6-phosphate amidotransferase 2 gene promoter. Gene **261**, 329-336. (10.1016/s0378-1119(00)00497-2) 11167021

[71] Zhang R, Lahens NF, Ballance HI, Hughes ME, Hogenesch JB. 2014 A circadian gene expression atlas in mammals: implications for biology and medicine. Proc. Natl Acad. Sci. USA **111**, 16 219-16 224. (10.1073/pnas.1408886111) PMC4234565 25349387

[72] Atger F et al. 2015 Circadian and feeding rhythms differentially affect rhythmic mRNA transcription and translation in mouse liver. Proc. Natl Acad. Sci. USA **112**, E6579-E6588. (10.1073/pnas.1515308112) 26554015 PMC4664316

[73] Zhou J, Huynh QK, Hoffman RT, Crook ED, Daniels MC, Gulve EA, McClain DA. 1998 Regulation of glutamine:fructose-6-phosphate amidotransferase by cAMP-dependent protein kinase. Diabetes **47**, 1836-1840. (10.2337/diabetes.47.12.1836) 9836513

[74] Chang Q, Su K, Baker JR, Yang X, Paterson AJ, Kudlow JE. 2000 Phosphorylation of human glutamine:fructose-6-phosphate amidotransferase by cAMP-dependent protein kinase at serine 205 blocks the enzyme activity. J. Biol. Chem. **275**, 21 981-21 987. (10.1074/jbc.M001049200) 10806197

[75] Hu Y, Riesland L, Paterson AJ, Kudlow JE. 2004 Phosphorylation of mouse glutamine-fructose-6-phosphate amidotransferase 2 (GFAT2) by cAMP-dependent protein kinase increases the enzyme activity. J. Biol. Chem. **279**, 29 988-29 993. (10.1074/jbc.M401547200) 15133036

[76] Ruegenberg S, Mayr FAMC, Atanassov I, Baumann U, Denzel MS. 2021 Protein kinase A controls the hexosamine pathway by tuning the feedback inhibition of GFAT-1. Nat. Commun. **12**, 2176. (10.1038/s41467-021-22320-y) 33846315 PMC8041777

[77] Li X et al. 2017 Myeloid-derived cullin 3 promotes STAT3 phosphorylation by inhibiting OGT expression and protects against intestinal inflammation. J. Exp. Med. **214**, 1093-1109. (10.1084/jem.20161105) 28280036 PMC5379975

[78] Ishii T, Warabi E, Mann GE. 2019 Circadian control of BDNF-mediated Nrf2 activation in astrocytes protects dopaminergic neurons from ferroptosis. Free Radic. Biol. Med. **133**, 169-178. (10.1016/j.freeradbiomed.2018.09.002) 30189266

[79] Joshi A, Upadhyay KK, Vohra A, Shirsath K, Devkar R. 2021 Melatonin induces Nrf2-HO-1 reprogramming and corrections in hepatic core clock oscillations in non-alcoholic fatty liver disease. FASEB J. Off. Publ. Fed. Am. Soc. Exp. Biol. **35**, e21803. (10.1096/fj.202002556RRR) 34365685

[80] Wang J et al. 2021 Circadian clock gene BMAL1 reduces urinary calcium oxalate stones formation by regulating NRF2/HO-1 pathway. Life Sci. **265**, 118853. (10.1016/j.lfs.2020.118853) 33278384

[81] Muthusamy S, Hong KU, Dassanayaka S, Hamid T, Jones SP. 2015 E2F1 transcription factor regulates O-linked N-acetylglucosamine (O-GlcNAc) transferase and O-GlcNAcase expression. J. Biol. Chem. **290**, 31 013-31 024. (10.1074/jbc.M115.677534) PMC4692227 26527687

[82] Schick S, Becker K, Thakurela S, Fournier D, Hampel MH, Legewie S, Tiwari VK. 2016 Identifying novel transcriptional regulators with circadian expression. Mol. Cell. Biol. **36**, 545-558. (10.1128/MCB.00701-15) 26644408 PMC4751687

[83] Menet JS, Rodriguez J, Abruzzi KC, Rosbash M. 2012 Nascent-Seq reveals novel features of mouse circadian transcriptional regulation. eLife **1**, e00011. (10.7554/eLife.00011) 23150795 PMC3492862

[84] Zhang C et al. 2019 Hepatocyte nuclear factor 1 alpha (HNF1A) regulates transcription of *O*-GlcNAc transferase in a negative feedback mechanism. FEBS Lett. **593**, 1050-1060. (10.1002/1873-3468.13381) 30953348

[85] Yang Y, Yin X, Yang H, Xu Y. 2015 Histone demethylase LSD2 Acts as an E3 ubiquitin ligase and inhibits cancer cell growth through promoting proteasomal degradation of OGT. Mol. Cell **58**, 47-59. (10.1016/j.molcel.2015.01.038) 25773598

[86] Peng K et al. 2021 Regulation of O-linked N-acetyl glucosamine transferase (OGT) through E6 stimulation of the ubiquitin ligase activity of E6AP. Int. J. Mol. Sci. **22**, 10286. (10.3390/ijms221910286) 34638625 PMC8508608

[87] Berthier A et al. 2018 Combinatorial regulation of hepatic cytoplasmic signaling and nuclear transcriptional events by the OGT/REV-ERB*α* complex. Proc. Natl Acad. Sci. USA **115**, E11 033-E11 042. (10.1073/pnas.1805397115) PMC6255172 30397120

[88] Kondratov RV, Antoch MP. 2007 Circadian proteins in the regulation of cell cycle and genotoxic stress responses. Trends Cell Biol. **17**, 311-317. (10.1016/j.tcb.2007.07.001) 17644383

[89] Li Z, Li X, Nai S, Geng Q, Liao J, Xu X, Li J. 2017 Checkpoint kinase 1–induced phosphorylation of O-linked β-N-acetylglucosamine transferase regulates the intermediate filament network during cytokinesis. J. Biol. Chem. **292**, 19 548-19 555. (10.1074/jbc.M117.811646) PMC5712597 29021254

[90] Deng X, Yi X, Huang D, Liu P, Chen L, Du Y, Hao L. 2020 ROCK2 mediates osteosarcoma progression and TRAIL resistance by modulating O-GlcNAc transferase degradation. Am. J. Cancer Res. **10**, 781-798. 32266091 PMC7136927

[91] Janich P, Toufighi K, Solanas G, Luis NM, Minkwitz S, Serrano L, Lehner B, Benitah SA. 2013 Human epidermal stem cell function is regulated by circadian oscillations. Cell Stem Cell **13**, 745-753. (10.1016/j.stem.2013.09.004) 24120744

[92] Cheung WD, Sakabe K, Housley MP, Dias WB, Hart GW. 2008 O-linked β-N-acetylglucosaminyltransferase substrate specificity is regulated by myosin phosphatase targeting and other interacting proteins. J. Biol. Chem. **283**, 33 935-33 941. (10.1074/jbc.M806199200) PMC2590692 18840611

[93] Zhang Z, Tan EP, VandenHull NJ, Peterson KR, Slawson C. 2014 O-GlcNAcase expression is sensitive to changes in O-GlcNAc homeostasis. Front. Endocrinol. **5**, 206. (10.3389/fendo.2014.00206) PMC4249489 25520704

[94] Petrus P et al. 2022 The central clock suffices to drive the majority of circulatory metabolic rhythms. Sci. Adv. **8**, eabo2896. (10.1126/sciadv.abo2896) 35767612 PMC9242453

[95] Xin H et al. 2021 A multi-tissue multi-omics analysis reveals distinct kineztics in entrainment of diurnal transcriptomes by inverted feeding. iScience **24**, 102335. (10.1016/j.isci.2021.102335) 33889826 PMC8050734

[96] Rhoades SD, Nayak K, Zhang SL, Sehgal A, Weljie AM. 2018 Circadian- and light-driven metabolic rhythms in *Drosophila melanogaster*. J. Biol. Rhythms **33**, 126-136. (10.1177/0748730417753003) 29355066 PMC6692290

[97] Marshall S, Nadeau O, Yamasaki K. 2004 Dynamic actions of glucose and glucosamine on hexosamine biosynthesis in isolated adipocytes. J. Biol. Chem. **279**, 35 313-35 319. (10.1074/jbc.M404133200) 15199059

[98] Liu J, Marchase RB, Chatham JC. 2007 Increased *O*-GlcNAc levels during reperfusion lead to improved functional recovery and reduced calpain proteolysis. Am. J. Physiol.-Heart Circ. Physiol. **293**, H1391-H1399. (10.1152/ajpheart.00285.2007) 17573462 PMC2850209

[99] Grigorian A, Lee S-U, Tian W, Chen IJ, Gao G, Mendelsohn R, Dennis JW, Demetriou M. 2007 Control of T cell-mediated autoimmunity by metabolite flux to N-glycan biosynthesis. J. Biol. Chem. **282**, 20 027-20 035. (10.1074/jbc.M701890200) 17488719

[100] Nakajima K, Kitazume S, Angata T, Fujinawa R, Ohtsubo K, Miyoshi E, Taniguchi N. 2010 Simultaneous determination of nucleotide sugars with ion-pair reversed-phase HPLC. Glycobiology **20**, 865-871. (10.1093/glycob/cwq044) 20371511

[101] Wellen KE, Lu C, Mancuso A, Lemons JMS, Ryczko M, Dennis JW, Rabinowitz JD, Coller HA, Thompson CB. 2010 The hexosamine biosynthetic pathway couples growth factor-induced glutamine uptake to glucose metabolism. Genes Dev. **24**, 2784-2799. (10.1101/gad.1985910) 21106670 PMC3003197

[102] Palorini R, Cammarata FP, Balestrieri C, Monestiroli A, Vasso M, Gelfi C, Alberghina L, Chiaradonna F. 2013 Glucose starvation induces cell death in K-ras-transformed cells by interfering with the hexosamine biosynthesis pathway and activating the unfolded protein response. Cell Death Dis. **4**, e732. (10.1038/cddis.2013.257) 23868065 PMC3730427

[103] Abdel Rahman AM, Ryczko M, Pawling J, Dennis JW. 2013 Probing the hexosamine biosynthetic pathway in human tumor cells by multitargeted tandem mass spectrometry. ACS Chem. Biol. **8**, 2053-2062. (10.1021/cb4004173) 23875632

[104] Swamy M, Pathak S, Grzes KM, Damerow S, Sinclair LV, van Aalten DMF, Cantrell DA. 2016 Glucose and glutamine fuel protein O-GlcNAcylation to control T cell self-renewal and malignancy. Nat. Immunol. **17**, 712-720. (10.1038/ni.3439) 27111141 PMC4900450

[105] Vasconcelos-dos-Santos A et al. 2017 Hyperglycemia exacerbates colon cancer malignancy through hexosamine biosynthetic pathway. Oncogenesis **6**, e306. (10.1038/oncsis.2017.2) 28319096 PMC5533945

[106] Pham LV et al. 2016 Targeting the hexosamine biosynthetic pathway and O-linked N-acetylglucosamine cycling for therapeutic and imaging capabilities in diffuse large B-cell lymphoma. Oncotarget **7**, 80 599-80 611. (10.18632/oncotarget.12413) PMC5348344 27716624

[107] Hawkins M, Barzilai N, Liu R, Hu M, Chen W, Rossetti L. 1997 Role of the glucosamine pathway in fat-induced insulin resistance. J. Clin. Invest. **99**, 2173-2182. (10.1172/JCI119390) 9151789 PMC508047

[108] Li X et al. 2018 CirGRDB: a database for the genome-wide deciphering circadian genes and regulators. Nucleic Acids Res. **46**, D64-D70. (10.1093/nar/gkx944) 29059379 PMC5753205

[109] Rodriguez J, Tang C-HA, Khodor YL, Vodala S, Menet JS, Rosbash M. 2013 Nascent-Seq analysis of *Drosophila* cycling gene expression. Proc. Natl Acad. Sci. USA **110**, E275-E284. (10.1073/pnas.1219969110) 23297234 PMC3557077

[110] Mauvoisin D, Wang J, Jouffe C, Martin E, Atger F, Waridel P, Quadroni M, Gachon F, Naef F. 2014 Circadian clock-dependent and -independent rhythmic proteomes implement distinct diurnal functions in mouse liver. Proc. Natl Acad. Sci. USA **111**, 167-172. (10.1073/pnas.1314066111) 24344304 PMC3890886

[111] Yamazaki K. 2014 Glutamine–Fructose-6-Phosphate Transaminase 1,2 (GFPT1,2). In Handbook of glycosyltransferases and related genes (eds N Taniguchi, K Honke, M Fukuda, H Narimatsu, Y Yamaguchi, T Angata), pp. 1465-1479. Tokyo, Japan: Springer.

[112] Richards AM, Nicholls MG, Espiner EA, Ikram H, Cullens M, Hinton D. 1986 Diurnal patterns of blood pressure, heart rate and vasoactive hormones in normal man. Clin. Exp. Hypertens. A **8**, 153-166. (10.3109/10641968609074769) 3521953

[113] James LR, Ingram A, Ly H, Thai K, Cai L, Scholey JW. 2001 Angiotensin II activates the GFAT promoter in mesangial cells. Am. J. Physiol.-Ren. Physiol. **281**, F151-F162. (10.1152/ajprenal.2001.281.1.F151) 11399656

[114] Isobe S et al. 2016 Augmented circadian rhythm of the intrarenal renin–angiotensin systems in anti-thymocyte serum nephritis rats. Hypertens. Res. **39**, 312-320. (10.1038/hr.2015.151) 26739872

[115] Hughes ME, Hong H-K, Chong JL, Indacochea AA, Lee SS, Han M, Takahashi JS, Hogenesch JB. 2012 Brain-specific rescue of clock reveals system-driven transcriptional rhythms in peripheral tissue. PLoS Genet. **8**, e1002835. (10.1371/journal.pgen.1002835) 22844252 PMC3405989

[116] Hughes ME, DiTacchio L, Hayes KR, Vollmers C, Pulivarthy S, Baggs JE, Panda S, Hogenesch JB. 2009 Harmonics of circadian gene transcription in mammals. PLoS Genet. **5**, e1000442. (10.1371/journal.pgen.1000442) 19343201 PMC2654964

[117] Masri S et al. 2014 Partitioning circadian transcription by SIRT6 leads to segregated control of cellular metabolism. Cell **158**, 659-672. (10.1016/j.cell.2014.06.050) 25083875 PMC5472354

[118] Janich P, Arpat AB, Castelo-Szekely V, Lopes M, Gatfield D. 2015 Ribosome profiling reveals the rhythmic liver translatome and circadian clock regulation by upstream open reading frames. Genome Res. **25**, 1848-1859. (10.1101/gr.195404.115) 26486724 PMC4665006

[119] Yang G et al. 2016 Timing of expression of the core clock gene Bmal1 influences its effects on aging and survival. Sci. Transl. Med. **8**, 324ra16. (10.1126/scitranslmed.aad3305) PMC4870001 26843191

[120] Jouffe C, Cretenet G, Symul L, Martin E, Atger F, Naef F, Gachon F. 2013 The circadian clock coordinates ribosome biogenesis. PLoS Biol. **11**, e1001455. (10.1371/journal.pbio.1001455) 23300384 PMC3536797

[121] Kuintzle RC, Chow ES, Westby TN, Gvakharia BO, Giebultowicz JM, Hendrix DA. 2017 Circadian deep sequencing reveals stress-response genes that adopt robust rhythmic expression during aging. Nat. Commun. **8**, 14529. (10.1038/ncomms14529) 28221375 PMC5321795

[122] Terajima H, Yoshitane H, Ozaki H, Suzuki Y, Shimba S, Kuroda S, Iwasaki W, Fukada Y. 2017 ADARB1 catalyzes circadian A-to-I editing and regulates RNA rhythm. Nat. Genet. **49**, 146-151. (10.1038/ng.3731) 27893733

[123] Hornbeck PV, Zhang B, Murray B, Kornhauser JM, Latham V, Skrzypek E. 2015 PhosphoSitePlus, 2014: mutations, PTMs and recalibrations. Nucleic Acids Res. **43**, D512-D520. (10.1093/nar/gku1267) 25514926 PMC4383998

[124] Allison DF, Wamsley JJ, Kumar M, Li D, Gray LG, Hart GW, Jones DR, Mayo MW. 2012 Modification of RelA by O-linked N-acetylglucosamine links glucose metabolism to NF-κB acetylation and transcription. Proc. Natl Acad. Sci. USA **109**, 16 888-16 893. (10.1073/pnas.1208468109) PMC3479489 23027940

[125] Ma Z, Chalkley RJ, Vosseller K. 2017 Hyper-O-GlcNAcylation activates nuclear factor κ-light-chain-enhancer of activated B cells (NF-κB) signaling through interplay with phosphorylation and acetylation. J. Biol. Chem. **292**, 9150-9163. (10.1074/jbc.M116.766568) 28416608 PMC5454098

[126] Kronlage M et al. 2019 O-GlcNAcylation of histone deacetylase 4 protects the diabetic heart from failure. Circulation **140**, 580-594. (10.1161/CIRCULATIONAHA.117.031942) 31195810

[127] Fujiki R et al. 2011 GlcNAcylation of histone H2B facilitates its monoubiquitination. Nature **480**, 557-560. (10.1038/nature10656) 22121020 PMC7289526

[128] Ruan H-B, Nie Y, Yang X. 2013 Regulation of protein degradation by O-GlcNAcylation: crosstalk with ubiquitination. Mol. Cell. Proteomics MCP **12**, 3489-3497. (10.1074/mcp.R113.029751) 23824911 PMC3861702

[129] Wang Z, Gucek M, Hart GW. 2008 Cross-talk between GlcNAcylation and phosphorylation: site-specific phosphorylation dynamics in response to globally elevated *O*-GlcNAc. Proc. Natl Acad. Sci. USA **105**, 13 793-13 798. (10.1073/pnas.0806216105) 18779572 PMC2544533

[130] Trinidad JC, Barkan DT, Thalhammer A, Sali A. 2012 Global identification and characterization of both O-GlcNAcylation and phosphorylation at the murine synapse. Mol. Cell. Proteomics **11**, 215-229. (10.1074/mcp.O112.018366) 22645316 PMC3412957

[131] Fan Z, Li J, Liu T, Zhang Z, Qin W, Qian X. 2021 A new tandem enrichment strategy for the simultaneous profiling of *O*-GlcNAcylation and phosphorylation in RNA-binding proteome. Analyst **146**, 1188-1197. (10.1039/D0AN02305A) 33465208

[132] Yao H, Li A, Wang M. 2015 Systematic analysis and prediction of *In Situ* cross talk of O-GlcNAcylation and phosphorylation. BioMed Res. Int. **2015**, 279823. (10.1155/2015/279823) 26601103 PMC4639640

[133] Leney AC, El Atmioui D, Wu W, Ovaa H, Heck AJR. 2017 Elucidating crosstalk mechanisms between phosphorylation and O-GlcNAcylation. Proc. Natl Acad. Sci. USA **114**, E7255-E7261. (10.1073/pnas.1620529114) 28808029 PMC5584407

[134] Butkinaree C, Park K, Hart GW. 2010 O-linked β-N-acetylglucosamine (O-GlcNAc): extensive crosstalk with phosphorylation to regulate signaling and transcription in response to nutrients and stress. Biochim. Biophys. Acta **1800**, 96-106. (10.1016/j.bbagen.2009.07.018) 19647786 PMC2815129

[135] Schwein PA, Woo CM. 2020 The O-GlcNAc modification on kinases. ACS Chem. Biol. **15**, 602-617. (10.1021/acschembio.9b01015) 32155042 PMC7253032

[136] Dias WB, Cheung WD, Hart GW. 2012 O-GlcNAcylation of kinases. Biochem. Biophys. Res. Commun. **422**, 224-228. (10.1016/j.bbrc.2012.04.124) 22564745 PMC3387735

[137] Shi J, Wu S, Dai C, Li Y, Grundke-Iqbal I, Iqbal K, Liu F, Gong C-X. 2012 Diverse regulation of AKT and GSK-3*β* by O-GlcNAcylation in various types of cells. FEBS Lett. **586**, 2443-2450. (10.1016/j.febslet.2012.05.063) 22687243 PMC3407308

[138] Shi J, Gu J, Dai C, Gu J, Jin X, Sun J, Iqbal K, Liu F, Gong C-X. 2015 O-GlcNAcylation regulates ischemia-induced neuronal apoptosis through AKT signaling. Sci. Rep. **5**, 14500. (10.1038/srep14500) 26412745 PMC4585968

[139] Kazemi Z, Chang H, Haserodt S, McKen C, Zachara NE. 2010 O-Linked β-N-acetylglucosamine (O-GlcNAc) regulates stress-induced heat shock protein expression in a GSK-3β-dependent manner. J. Biol. Chem. **285**, 39 096-39 107. (10.1074/jbc.M110.131102) PMC2998145 20926391

[140] Inoue Y, Moriwaki K, Ueda Y, Takeuchi T, Higuchi K, Asahi M. 2018 Elevated O-GlcNAcylation stabilizes FOXM1 by its reduced degradation through GSK-3*β* inactivation in a human gastric carcinoma cell line, MKN45 cells. Biochem. Biophys. Res. Commun. **495**, 1681-1687. (10.1016/j.bbrc.2017.11.179) 29196265

[141] Yang Y et al. 2020 OGT suppresses S6K1-mediated macrophage inflammation and metabolic disturbance. Proc. Natl Acad. Sci. USA **117**, 16 616-16 625. (10.1073/pnas.1916121117) PMC7368321 32601203

[142] Pang Y et al. 2021 High fat activates O-GlcNAcylation and affects AMPK/ACC pathway to regulate lipid metabolism. Nutrients **13**, 1740. (10.3390/nu13061740) 34063748 PMC8223797

[143] Miura T, Kume M, Kawamura T, Yamamoto K, Hamakubo T, Nishihara S. 2018 O-GlcNAc on PKC*ζ* Inhibits the FGF4-PKCζ-MEK-ERK1/2 pathway via inhibition of PKC*ζ* phosphorylation in mouse embryonic stem cells. Stem Cell Rep. **10**, 272-286. (10.1016/j.stemcr.2017.11.007) PMC5768893 29249667

[144] Guo X, Deng Y, Zhan L, Shang J, Liu H. 2021 O-GlcNAcylation contributes to intermittent hypoxia-associated vascular dysfunction via modulation of MAPKs but not CaMKII pathways. Mol. Med. Rep. **24**, 744. (10.3892/mmr.2021.12384) 34435655 PMC8430318

[145] Tarrant MK et al. 2012 Regulation of CK2 by phosphorylation and O-GlcNAcylation revealed by semisynthesis. Nat. Chem. Biol. **8**, 262-269. (10.1038/nchembio.771) 22267120 PMC3288285

[146] Schwein PA, Ge Y, Yang B, D'Souza A, Mody A, Shen D, Woo CM. 2022 Writing and erasing O-GlcNAc on casein kinase 2 alpha alters the phosphoproteome. ACS Chem. Biol. **17**, 1111-1121. (10.1021/acschembio.1c00987) 35467332 PMC9647470

[147] Dong DL, Xu ZS, Chevrier MR, Cotter RJ, Cleveland DW, Hart GW. 1993 Glycosylation of mammalian neurofilaments. Localization of multiple O-linked N-acetylglucosamine moieties on neurofilament polypeptides L and M. J. Biol. Chem. **268**, 16 679-16 687. (10.1016/S0021-9258(19)85471-6) 8344946

[148] Gao Y, Wells L, Comer FI, Parker GJ, Hart GW. 2001 Dynamic O-Glycosylation of nuclear and cytosolic proteins. J. Biol. Chem. **276**, 9838-9845. (10.1074/jbc.M010420200) 11148210

[149] Akimoto Y, Comer FI, Cole RN, Kudo A, Kawakami H, Hirano H, Hart GW. 2003 Localization of the O-GlcNAc transferase and O-GlcNAc-modified proteins in rat cerebellar cortex. Brain Res. **966**, 194-205. (10.1016/S0006-8993(02)04158-6) 12618343

[150] Yang X, Zhang F, Kudlow JE. 2002 Recruitment of O-GlcNAc transferase to promoters by corepressor mSin3A: coupling protein O-GlcNAcylation to transcriptional repression. Cell **110**, 69-80. (10.1016/s0092-8674(02)00810-3) 12150998

[151] Hahne H, Gholami AM, Kuster B. 2012 Discovery of O-GlcNAc-modified proteins in published large-scale proteome data. Mol. Cell. Proteomics MCP **11**, 843-850. (10.1074/mcp.M112.019463) 22661428 PMC3494142

[152] Reinke H, Asher G. 2019 Crosstalk between metabolism and circadian clocks. Nat. Rev. Mol. Cell Biol. **20**, 227-241. (10.1038/s41580-018-0096-9) 30635659

[153] Khidekel N, Ficarro SB, Peters EC, Hsieh-Wilson LC. 2004 Exploring the *O*-GlcNAc proteome: direct identification of *O*-GlcNAc-modified proteins from the brain. Proc. Natl Acad. Sci. USA **101**, 13 132-13 137. (10.1073/pnas.0403471101) PMC516536 15340146

[154] Khidekel N et al. 2007 Probing the dynamics of O-GlcNAc glycosylation in the brain using quantitative proteomics. Nat. Chem. Biol. **3**, 339-348. (10.1038/nchembio881) 17496889

[155] Tramutola A et al. 2018 Proteomic identification of altered protein O-GlcNAcylation in a triple transgenic mouse model of Alzheimer's disease. Biochim. Biophys. Acta **1864**, 3309-3321. (10.1016/j.bbadis.2018.07.017) 30031227

[156] Alfaro JF et al. 2012 Tandem mass spectrometry identifies many mouse brain *O*-GlcNAcylated proteins including EGF domain-specific *O*-GlcNAc transferase targets. Proc. Natl Acad. Sci. USA **109**, 7280-7285. (10.1073/pnas.1200425109) 22517741 PMC3358849

[157] Huynh VN et al. 2021 Defining the dynamic regulation of O-GlcNAc proteome in the mouse cortex—the O-GlcNAcylation of synaptic and trafficking proteins related to neurodegenerative diseases. Front. Aging **2**, 757801. (10.3389/fragi.2021.757801) 35822049 PMC9261315

[158] Wells L, Vosseller K, Cole RN, Cronshaw JM, Matunis MJ, Hart GW. 2002 Mapping sites of O-GlcNAc modification using affinity tags for serine and threonine post-translational modifications. Mol. Cell. Proteomics **1**, 791-804. (10.1074/mcp.M200048-MCP200) 12438562

[159] Wang S et al. 2017 Quantitative proteomics identifies altered O-GlcNAcylation of structural, synaptic and memory-associated proteins in Alzheimer's disease. J. Pathol. **243**, 78-88. (10.1002/path.4929) 28657654 PMC5647145

[160] Cieniewski-Bernard C, Bastide B, Lefebvre T, Lemoine J, Mounier Y, Michalski J-C. 2004 Identification of O-linked N-acetylglucosamine proteins in rat skeletal muscle using two-dimensional gel electrophoresis and mass spectrometry. Mol. Cell. Proteomics **3**, 577-585. (10.1074/mcp.M400024-MCP200) 14985449

[161] Deracinois B, Camoin L, Lambert M, Boyer J-B, Dupont E, Bastide B, Cieniewski-Bernard C. 2018 O-GlcNAcylation site mapping by (azide-alkyne) click chemistry and mass spectrometry following intensive fractionation of skeletal muscle cells proteins. J. Proteomics **186**, 83-97. (10.1016/j.jprot.2018.07.005) 30016717

[162] Prisco SZ et al. 2020 Excess protein O-GlcNAcylation links metabolic derangements to right ventricular dysfunction in pulmonary arterial hypertension. Int. J. Mol. Sci. **21**, 7278. (10.3390/ijms21197278) 33019763 PMC7582480

[163] Hahne H, Sobotzki N, Nyberg T, Helm D, Borodkin VS, van Aalten DMF, Agnew B, Kuster B. 2013 Proteome wide purification and identification of *O*-GlcNAc-modified proteins using click chemistry and mass spectrometry. J. Proteome Res. **12**, 927-936. (10.1021/pr300967y) 23301498 PMC4946622

[164] Zhu Y, Willems LI, Salas D, Cecioni S, Wu WB, Foster LJ, Vocadlo DJ. 2020 Tandem bioorthogonal labeling uncovers endogenous cotranslationally *O*-GlcNAc modified nascent proteins. J. Am. Chem. Soc. **142**, 15 729-15 739. (10.1021/jacs.0c04121) 32870666

[165] Teo CF, Ingale S, Wolfert MA, Elsayed GA, Nöt LG, Chatham JC, Wells L, Boons G-J. 2010 Glycopeptide-specific monoclonal antibodies suggest new roles for O-GlcNAc. Nat. Chem. Biol. **6**, 338-343. (10.1038/nchembio.338) 20305658 PMC2857662

[166] Shen B, Zhang W, Shi Z, Tian F, Deng Y, Sun C, Wang G, Qin W, Qian X. 2017 A novel strategy for global mapping of O-GlcNAc proteins and peptides using selective enzymatic deglycosylation, HILIC enrichment and mass spectrometry identification. Talanta **169**, 195-202. (10.1016/j.talanta.2017.03.049) 28411811

[167] Lopez Aguilar A, Gao Y, Hou X, Lauvau G, Yates JR, Wu P. 2017 Profiling of protein *O*-GlcNAcylation in Murine CD8 ^+^ effector- and memory-like T cells. ACS Chem. Biol. **12**, 3031-3038. (10.1021/acschembio.7b00869) 29125738 PMC5931335

[168] Lund PJ, Elias JE, Davis MM. 2016 Global analysis of *O*-GlcNAc glycoproteins in activated human T cells. J. Immunol. **197**, 3086-3098. (10.4049/jimmunol.1502031) 27655845 PMC5055199

[169] Woo CM, Lund PJ, Huang AC, Davis MM, Bertozzi CR, Pitteri SJ. 2018 Mapping and quantification of over 2000 O-linked glycopeptides in activated human T cells with isotope-targeted glycoproteomics (Isotag). Mol. Cell. Proteomics **17**, 764-775. (10.1074/mcp.RA117.000261) 29351928 PMC5880114

[170] Sprung R, Nandi A, Chen Y, Kim SC, Barma D, Falck JR, Zhao Y. 2005 Tagging-via-substrate strategy for probing O-GlcNAc modified proteins. J. Proteome Res. **4**, 950-957. (10.1021/pr050033j) 15952742

[171] Nagel AK, Schilling M, Comte-Walters S, Berkaw MN, Ball LE. 2013 Identification of O-linked N-acetylglucosamine (O-GlcNAc)-modified osteoblast proteins by electron transfer dissociation tandem mass spectrometry reveals proteins critical for bone formation. Mol. Cell. Proteomics **12**, 945-955. (10.1074/mcp.M112.026633) 23443134 PMC3617341

[172] Liu J et al. 2021 Quantitative chemoproteomics reveals O-GlcNAcylation of cystathionine γ-lyase (CSE) represses trophoblast syncytialization. Cell Chem. Biol. **28**, 788-801.e5. (10.1016/j.chembiol.2021.01.024) 33626323

[173] Zaro BW, Yang Y-Y, Hang HC, Pratt MR. 2011 Chemical reporters for fluorescent detection and identification of O-GlcNAc-modified proteins reveal glycosylation of the ubiquitin ligase NEDD4-1. Proc. Natl Acad. Sci. USA **108**, 8146-8151. (10.1073/pnas.1102458108) 21540332 PMC3100932

[174] Okamoto-Uchida Y, Izawa J, Nishimura A, Hattori A, Suzuki N, Hirayama J. 2019 Post-translational modifications are required for circadian clock regulation in vertebrates. Curr. Genomics **20**, 332-339. (10.2174/1389202919666191014094349) 32476990 PMC7235395

[175] Mauvoisin D. 2019 Circadian rhythms and proteomics: it's all about posttranslational modifications! Wiley Interdiscip. Rev. Syst. Biol. Med. **11**, e1450. (10.1002/wsbm.1450) 31034157

[176] Srikanta SB, Cermakian N. 2021 To Ub or not to Ub: regulation of circadian clocks by ubiquitination and deubiquitination. J. Neurochem. **157**, 11-30. (10.1111/jnc.15132) 32717140

[177] Edery I, Zwiebel LJ, Dembinska ME, Rosbash M. 1994 Temporal phosphorylation of the *Drosophila* period protein. Proc. Natl Acad. Sci. USA **91**, 2260-2264. (10.1073/pnas.91.6.2260) 8134384 PMC43350

[178] Narasimamurthy R, Virshup DM. 2021 The phosphorylation switch that regulates ticking of the circadian clock. Mol. Cell **81**, 1133-1146. (10.1016/j.molcel.2021.01.006) 33545069

[179] Mendoza-Viveros L, Bouchard-Cannon P, Hegazi S, Cheng AH, Pastore S, Cheng H-YM. 2017 Molecular modulators of the circadian clock: lessons from flies and mice. Cell. Mol. Life Sci. **74**, 1035-1059. (10.1007/s00018-016-2378-8) 27689221 PMC11107503

[180] Cai YD et al. 2021 CK2 inhibits TIMELESS nuclear export and modulates CLOCK transcriptional activity to regulate circadian rhythms. Curr. Biol. **31**, 502-514.e7. (10.1016/j.cub.2020.10.061) 33217322 PMC7878342

[181] Yang X, Qian K. 2017 Protein O-GlcNAcylation: emerging mechanisms and functions. Nat. Rev. Mol. Cell Biol. **18**, 452-465. (10.1038/nrm.2017.22) 28488703 PMC5667541

[182] Gonzalez-Rellan MJ, Fondevila MF, Dieguez C, Nogueiras R. 2022 O-GlcNAcylation: a sweet hub in the regulation of glucose metabolism in health and disease. Front. Endocrinol. **13**, 873513. (10.3389/fendo.2022.873513) PMC9072661 35527999

[183] Lee JB, Pyo K-H, Kim HR. 2021 Role and function of O-GlcNAcylation in cancer. Cancers **13**, 5365. (10.3390/cancers13215365) 34771527 PMC8582477

[184] Sun L, Lv S, Song T. 2021 O-GlcNAcylation links oncogenic signals and cancer epigenetics. Discov. Oncol. **12**, 54. (10.1007/s12672-021-00450-5) 35201498 PMC8777512

[185] Yang R, Wang L, Wu Z, Yin Y, Jiang S-W. 2022 How nanotechniques could vitalize the O-GlcNAcylation-targeting approach for cancer therapy. Int. J. Nanomedicine **17**, 1829-1841. (10.2147/IJN.S360488) 35498390 PMC9049135

[186] Selvan N et al. 2017 A mutant O-GlcNAcase enriches *Drosophila* developmental regulators. Nat. Chem. Biol. **13**, 882-887. (10.1038/nchembio.2404) 28604694 PMC7611224

[187] Qin W, Xie Z, Wang J, Ou G, Wang C, Chen X. 2020 Chemoproteomic profiling of O-GlcNAcylation in *Caenorhabditis elegans*. Biochemistry **59**, 3129-3134. (10.1021/acs.biochem.9b00622) 31682414

[188] Akan I, Halim A, Vakhrushev SY, Clausen H, Hanover JA. 2021 *Drosophila* O-GlcNAcase mutants reveal an expanded glycoproteome and novel growth and longevity phenotypes. Cells **10**, 1026. (10.3390/cells10051026) 33925313 PMC8145559

[189] Ma J, Liu T, Wei A-C, Banerjee P, O'Rourke B, Hart GW. 2015 O-GlcNAcomic profiling identifies widespread O-linked β-N-acetylglucosamine modification (O-GlcNAcylation) in oxidative phosphorylation system regulating cardiac mitochondrial function. J. Biol. Chem. **290**, 29 141-29 153. (10.1074/jbc.M115.691741) PMC4705920 26446791

[190] Ma J et al. 2016 Comparative proteomics reveals dysregulated mitochondrial O-GlcNAcylation in diabetic hearts. J. Proteome Res. **15**, 2254-2264. (10.1021/acs.jproteome.6b00250) 27213235 PMC7814404

[191] Cao W, Cao J, Huang J, Yao J, Yan G, Xu H, Yang P. 2013 Discovery and confirmation of O-glcnacylated proteins in rat liver mitochondria by combination of mass spectrometry and immunological methods. PLoS ONE **8**, e76399. (10.1371/journal.pone.0076399) 24098488 PMC3788734

[192] Ramirez-Correa GA, Jin W, Wang Z, Zhong X, Gao WD, Dias WB, Vecoli C, Hart GW, Murphy AM. 2008 *O*-Linked GlcNAc modification of cardiac myofilament proteins: a novel regulator of myocardial contractile function. Circ. Res. **103**, 1354-1358. (10.1161/CIRCRESAHA.108.184978) 18988896 PMC2615199

[193] Vosseller K et al. 2006 O-linked N-acetylglucosamine proteomics of postsynaptic density preparations using lectin weak affinity chromatography and mass spectrometry. Mol. Cell. Proteomics **5**, 923-934. (10.1074/mcp.T500040-MCP200) 16452088

[194] Chalkley RJ, Thalhammer A, Schoepfer R, Burlingame AL. 2009 Identification of protein O-GlcNAcylation sites using electron transfer dissociation mass spectrometry on native peptides. Proc. Natl Acad. Sci. USA **106**, 8894-8899. (10.1073/pnas.0900288106) 19458039 PMC2690010

[195] Zeidan Q, Wang Z, De Maio A, Hart GW. 2010 *O*-GlcNAc cycling enzymes associate with the translational machinery and modify core ribosomal proteins. Mol. Biol. Cell **21**, 1922-1936. (10.1091/mbc.e09-11-0941) 20410138 PMC2883937

[196] Myers SA, Panning B, Burlingame AL. 2011 Polycomb repressive complex2 is necessary for the normal site-specific *O*-GlcNAc distribution in mouse embryonic stem cells. Proc. Natl Acad. Sci. USA **108**, 9490-9495. (10.1073/pnas.1019289108) 21606357 PMC3111310

[197] Lee A, Miller D, Henry R, Paruchuri VDP, O'Meally RN, Boronina T, Cole RN, Zachara NE. 2016 Combined antibody/lectin enrichment identifies extensive changes in the *O-*GlcNAc sub-proteome upon oxidative stress. J. Proteome Res. **15**, 4318-4336. (10.1021/acs.jproteome.6b00369) 27669760 PMC8132933

[198] Li J et al. 2019 An isotope-coded photocleavable probe for quantitative profiling of protein *O*-GlcNAcylation. ACS Chem. Biol. **14**, 4-10. (10.1021/acschembio.8b01052) 30620550

[199] Lin C-H, Liao C-C, Wang S-Y, Peng C-Y, Yeh Y-C, Chen M-Y, Chou T-Y. 2022 Comparative O-GlcNAc proteomic analysis reveals a role of O-GlcNAcylated SAM68 in lung cancer aggressiveness. Cancers **14**, 243. (10.3390/cancers14010243) 35008409 PMC8749979

[200] Drougat L, Olivier-Van Stichelen S, Mortuaire M, Foulquier F, Lacoste A-S, Michalski J-C, Lefebvre T, Vercoutter-Edouart A-S. 2012 Characterization of O-GlcNAc cycling and proteomic identification of differentially O-GlcNAcylated proteins during G1/S transition. Biochim. Biophys. Acta **1820**, 1839-1848. (10.1016/j.bbagen.2012.08.024) 22967762

[201] Liu J, Hao Y, He Y, Li X, Sun D, Zhang Y, Yang P-Y, Chen X. 2021 Quantitative and site-specific chemoproteomic profiling of protein O-GlcNAcylation in the cell cycle. ACS Chem. Biol. **16**, 1917-1923. (10.1021/acschembio.1c00301) 34161081

[202] Fleischer JG, Das SK, Bhapkar M, Manoogian ENC, Panda S. 2022 Associations between the timing of eating and weight-loss in calorically restricted healthy adults: findings from the CALERIE study. Exp. Gerontol. **165**, 111837. (10.1016/j.exger.2022.111837) 35598698

[203] Wilkinson MJ et al. 2020 Ten-hour time-restricted eating reduces weight, blood pressure, and atherogenic lipids in patients with metabolic syndrome. Cell Metab. **31**, 92-104.e5. (10.1016/j.cmet.2019.11.004) 31813824 PMC6953486

[204] Pendergast JS, Branecky KL, Yang W, Ellacott KLJ, Niswender KD, Yamazaki S. 2013 High-fat diet acutely affects circadian organisation and eating behavior. Eur. J. Neurosci. **37**, 1350-1356. (10.1111/ejn.12133) 23331763 PMC3645495

[205] Branecky KL, Niswender KD, Pendergast JS. 2015 Disruption of daily rhythms by high-fat diet is reversible. PLoS ONE **10**, e0137970. (10.1371/journal.pone.0137970) 26366733 PMC4569368

[206] Figlia G, Willnow P, Teleman AA. 2020 Metabolites regulate cell signaling and growth via covalent modification of proteins. Dev. Cell **54**, 156-170. (10.1016/j.devcel.2020.06.036) 32693055

[207] Bludau I, Willems S, Zeng W-F, Strauss MT, Hansen FM, Tanzer MC, Karayel O, Schulman BA, Mann M. 2022 The structural context of posttranslational modifications at a proteome-wide scale. PLoS Biol. **20**, e3001636. (10.1371/journal.pbio.3001636) 35576205 PMC9135334

